# The Synthesis of a Bis(thiosemicarbazone) Macrocyclic Ligand and the Mn(II), Co(II), Zn(II) and ^68^Ga(III) Complexes

**DOI:** 10.3390/molecules26123646

**Published:** 2021-06-15

**Authors:** Melyssa L. Grieve, Patrick R. W. J. Davey, Craig M. Forsyth, Brett M. Paterson

**Affiliations:** 1 School of Chemistry, Monash University, Clayton, VIC 3800, Australia; melyssa.grieve1@monash.edu (M.L.G.); patrick.davey1@monash.edu (P.R.W.J.D.); craig.forsyth@monash.edu (C.M.F.); 2 Monash Biomedical Imaging, Monash University, Clayton, VIC 3800, Australia

**Keywords:** thiosemicarbazone, macrocycle, transition metal, cyclen, gallium-68, coordination chemistry, radiochemistry

## Abstract

A 1,4,7,10-tetraazacyclododecane (cyclen) variant bearing two thiosemicarbazone pendant groups has been prepared. The ligand forms complexes with Mn^2+^, Co^2+^ and Zn^2+^. X-ray crystallography of the Mn^2+^, Co^2+^ and Zn^2+^ complexes showed that the ligand provides a six-coordinate environment for the metal ions. The Mn^2+^ and Zn^2+^ complexes exist in the solid state as racemic mixtures of the Δ(δ,δ,δ,δ)/Λ(λ,λ,λ,λ) and Δ(λ,λ,λ,λ)/Λ(δ,δ,δ,δ) diastereomers, and the Co^2+^ complex exists as the Δ(δ,δ,δ,δ)/Λ(λ,λ,λ,λ) and Δ(λ,λ,λ,δ)/Λ(δ,δ,δ,λ) diastereomers. Density functional theory calculations indicated that the relative energies of the diastereomers are within 10 kJ mol^−1^. Magnetic susceptibility of the complexes indicated that both the Mn^2+^ and Co^2+^ ions are high spin. The ligand was radiolabelled with gallium-68, in the interest of developing new positron emission tomography imaging agents, which produced a single species in high radiochemical purity (>95%) at 90 °C for 10 min.

## 1. Introduction

Thiosemicarbazone functional groups are versatile N,S donors that can coordinate metal ions as neutral or anionic ligands, with the resulting complexes displaying diverse coordination chemistry. In general, thiosemicarbazones are easy to synthesise by way of a condensation reaction between a thiosemicarbazide and an aldehyde or ketone. The structures can be modified in multiple ways, allowing for the generation of tri-, tetra-, penta- or even hexadentate ligands, as well as multinuclear complexes and coordination polymers [1,2,3,4,5,6,7,8,9]. Modifications to the thiosemicarbazone substituents can also result in dramatic changes to the structural, physical and biological properties of the metal complexes [10,11].

Hybrid ligands containing thiosemicarbazone groups and additional donor atoms can introduce modifiable properties to the complexes for a variety of biological applications [12,13,14]. *N*-heterocyclic thiosemicarbazones have been investigated for their pharmacological properties, which have shown that the metal complexes can display bioactivities which differ from those of either the ligand or the metal ion [15,16,17,18]. Lipophilic Mn^2+^, Zn^2+^ and Ga^3+^ complexes have demonstrated anti-tumour activity due to their ability to facilitate intracellular delivery of the free ligand upon metal dissociation or transmetallation to the Fe^3+^ or Cu^2+^ complexes [19,20,21,22,23,24]. Cobalt thiosemicarbazonato complexes have been isolated in the Co^2+^ and Co^3+^ oxidation states and have been investigated as redox-active prodrugs for hypoxia targeting and as anti-bacterial and anti-cancer agents [25,26,27]. Zinc ions have been found to play an important role in medicine, with compounds developed for treating neurodegeneration as well as anti-diabetic, anti-tumour, anti-bacterial, anti-microbial and anti-inflammatory agents [16,28,29,30,31,32,33,34].

Radioactive metal complexes of bis(thiosemicarbazonato) ligands derived from 1,2-diones have been of interest for diagnostic and therapeutic applications. The ligands form complexes with positron-emitting isotopes of copper such as copper-64 (^64^Cu, t_1/2_ = 12.7 h) that have been investigated as perfusion and hypoxia imaging agents and as probes for measuring altered copper trafficking in the brain during Alzheimer’s disease [35,36,37,38]. Copper complexes of thiosemicarbazone-pyridylhydrazone hybrid ligands, incorporating a targeting group within the chelate domain, demonstrate blood–brain barrier permeability and an ability to bind to amyloid-β plaques in vitro, and have the potential to assist with Alzheimer’s disease diagnosis [39,40]. Bis(thiosemicarbazones) have also gained attention as bifunctional chelators for labelling small peptides with radioactive isotopes of copper due to their fast radiolabelling kinetics under ambient conditions, and peptide coupling routes by way of ligands with pendant amine or carboxylic acid groups [41,42,43].

The positron emitter gallium-68 (^68^Ga, t_1/2_ = 68 min) is readily obtained from a ^68^Ge/^68^Ga generator from which it is eluted as a [^68^Ga][GaCl_4_]^-^ solution in aqueous acidic solution and is increasingly utilised for positron emission tomography (PET) [44]. The ^68^Ga^3+^ ion, administered as the citrate and nitrate salts, has been used to image tumours, inflammation and infection [45]. Incorporating the radionuclide into a stable bifunctional chelator is a method that allows for effective targeting to a wide range of disease states such as prostate cancer and neuroendocrine tumours [46,47,48]. Interest in the development of chelating agents for ^68^Ga^3+^ has been ongoing, mostly due to the requirements for fast radiolabelling in high yields at moderate temperatures and in vivo stability [49,50].

Macrocyclic polyamines such as 1,4,7,10-tetraazacyclododecane (cyclen) have been utilised in materials chemistry, medicinal chemistry, bioinorganic chemistry and chemical biology [51,52,53,54]. The metal complexes of macrocyclic polyamines have been vital to the development of radiopharmaceuticals owing to the higher kinetic inertness and thermodynamic stability in comparison to metal complexes of acyclic ligands [55,56,57,58]. The modifiable structures and the versatile chemistry of macrocyclic polyamines make them attractive scaffolds for generating hybrid ligands. These ligands incorporate various functional groups in an effort to assemble metal complexes suitable for a multitude of applications. The chelator 1,4,7,10-tetraazacyclododecane-1,4,7,10-tetraacetic acid (DOTA) is widely utilised to produce Gd^3+^ and Mn^2+^ magnetic resonance contrast agents and ^68^Ga^3+^, ^64^Cu^2+^ and ^177^Lu^3+^ radiopharmaceuticals [58,59,60,61,62].

Previously, we have used the tetraazamacrocyclic framework of cyclen to integrate two pendant semicarbazone groups to produce an octadentate chelator for Pb^2+^ and Bi^3+^ radionuclides [63]. In this work, we investigated the coordination chemistry of a tetraazamacrocycle incorporating thiosemicarbazone pendant groups with the transition metals Mn^2+^, Co^2+^ and Zn^2+^. Thiosemicarbazone complexes of ^68^Ga^3+^ and the gamma emitting radioisotope ^67^Ga^3+^ have been prepared previously, which prompted an investigation of the ^68^Ga^3+^ radiolabelling properties of the new chelator [64,65,66,67].

## 2. Results and Discussion

### 2.1. Synthesis of H_2_**L** and the Manganese(II), Cobalt(II) and Zinc(II) Complexes

**Scheme 1:**
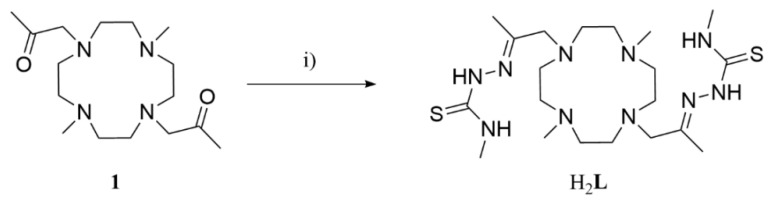
The synthetic route for the synthesis of H_2_**L**. i) 4-methyl-3-thiosemicarbazide, acetic acid, ethanol, rt, 48 h.

The synthesis of the diketone 1,1′-(4,10-dimethyl-1,4,7,10-tetraazacyclododecane-1,7-diyl)bis(propan-2-one) (**1**) has been previously reported [63]. The synthesis of H_2_**L** involves a condensation reaction between the ketone groups of **1** and two equivalents of 4-methyl-3-thiosemicarbazide (Scheme 1).

H_2_**L** was characterised by ^1^H and ^13^C{^1^H} NMR spectroscopy. Variable-temperature ^1^H NMR demonstrated sharpening of the macrocyclic CH_2_ proton signals (δ = 2.70–2.85 ppm) as the temperature increased, which suggested fluxionality (Figure S3) [68]. High-resolution mass spectrometry analysis showed an isotopic pattern at *m/z* = 487.31987 corresponding to the [H_2_**L** + H]^+^ cation (Figure S7). Analytical-HPLC indicated the presence of a single compound (*R*_T_ = 5.27 min) (Figure S11).

**Scheme 2:**
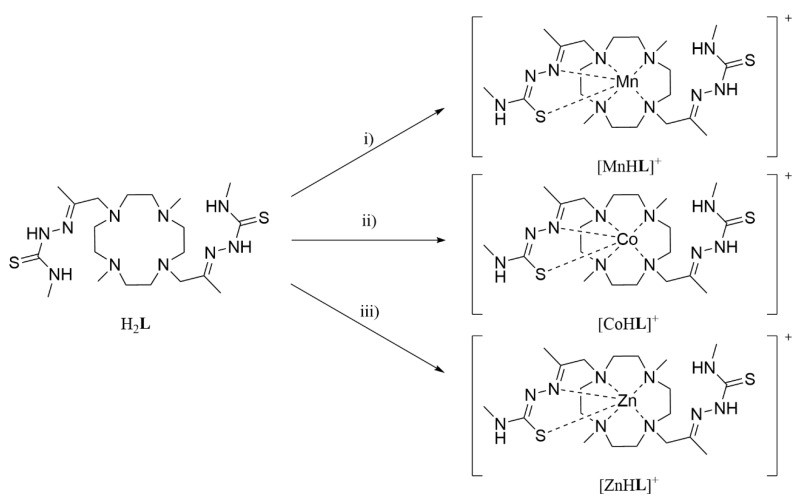
Synthesis of [MnH**L**](BPh_4_), [CoH**L**](BPh_4_) and [ZnH**L**](BPh_4_). i) Mn(OAc)_2_·4H_2_O, methanol, rt, NaBPh_4_, 24 h; ii) Co(OAc)_2_·4H_2_O, methanol, rt, NaBPh_4_, 24 h; iii) Zn(OAc)_2_·2H_2_O, methanol, rt, NaBPh_4_, 24 h.

The addition of the metal acetate salts to a suspension of H_2_**L** in methanolic solution gave the complexes [MnH**L**]^+^, [CoH**L**]^+^ and [ZnH**L**]^+^ (Scheme 2). Addition of NaBPh_4_ resulted in precipitation of the complexes as the tetraphenylborate salts. High-resolution mass spectrometry analysis showed isotopic patterns at *m/z* = 540.2335, 544.2298 and 549.2249 attributed to the [MnH**L**]^+^, [CoH**L**]^+^ and [ZnH**L**]^+^ complex cations, respectively (Figures S8–S10). Analytical-HPLC showed the presence of the compounds at retention times of 6.06, 6.05 and 6.01 min attributed to the [MnH**L**](BPh_4_), [CoH**L**](BPh_4_) and [ZnH**L**](BPh_4_) complexes, respectively (Figures S12–S14). Single crystals of the tetraphenylborate coordination compounds were obtained by slow diffusion of diethyl ether into solutions of the compounds in acetone.

### 2.2. NMR Studies

**Figure 1:**
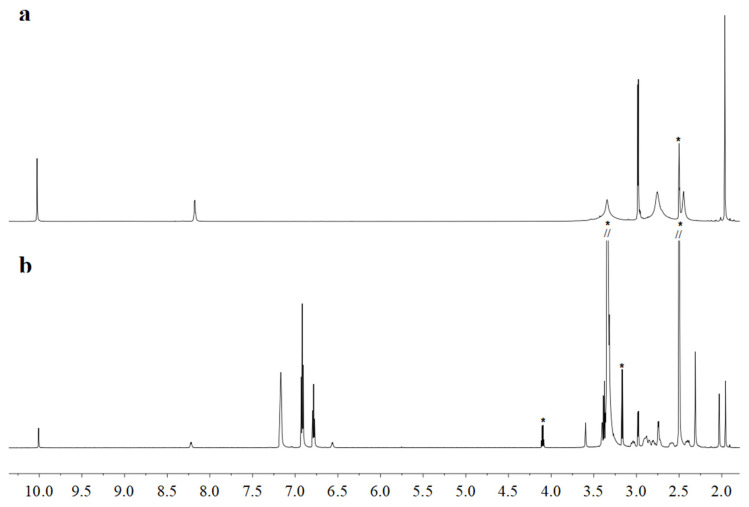
(**a**) The ^1^H NMR spectrum of H_2_**L** in DMSO-*d*_6_ at 25 °C. (**b**) The ^1^H NMR spectrum of [ZnH**L**](BPh_4_) in DMSO-*d*_6_ at 25 °C. Residual solvent peaks are marked with an asterisk.

The complex [ZnH**L**](BPh_4_) was characterised by ^1^H and ^13^C{^1^H} NMR spectroscopy. The presence of one equivalent of tetraphenylborate and a single hydrazinic nitrogen proton (δ = 10.01 ppm) indicated deprotonation of the ligand at the hydrazinic nitrogen of the coordinated pendant arm. The ligand resonances in the ^1^H NMR spectrum for the thiosemicarbazonato coordinated pendant arm of [ZnH**L**](BPh_4_) have shifted compared to the spectrum for H_2_**L** and the non-coordinated arm (Figure 1). For example, the methyl group in the pendant arm at δ = 1.96 ppm in the ^1^H NMR spectrum of H_2_**L** splits into two peaks at δ = 1.96 and 2.03 ppm for the complex. The two methyl groups attached to the macrocycle shifted from δ = 2.45 ppm for H_2_**L** to δ = 2.31 ppm for [ZnH**L**](BPh_4_). A significant shift was observed for the terminal NH proton of the thiosemicarbazonato arm from δ = 8.22 ppm to 6.56 ppm. Although the signals due to the protons of the macrocycle of [ZnH**L**](BPh_4_) (δ = 2.40–3.04 ppm) are somewhat broad, variable-temperature analysis of the ^1^H NMR spectra indicated that, unlike H_2_**L**, this was not temperature dependent (Figure S6).

### 2.3. X-ray Crystal Structures of [MnH**L**](BPh_4_), [CoH**L**](BPh_4_) and [ZnH**L**](BPh_4_)

**Figure 2:**
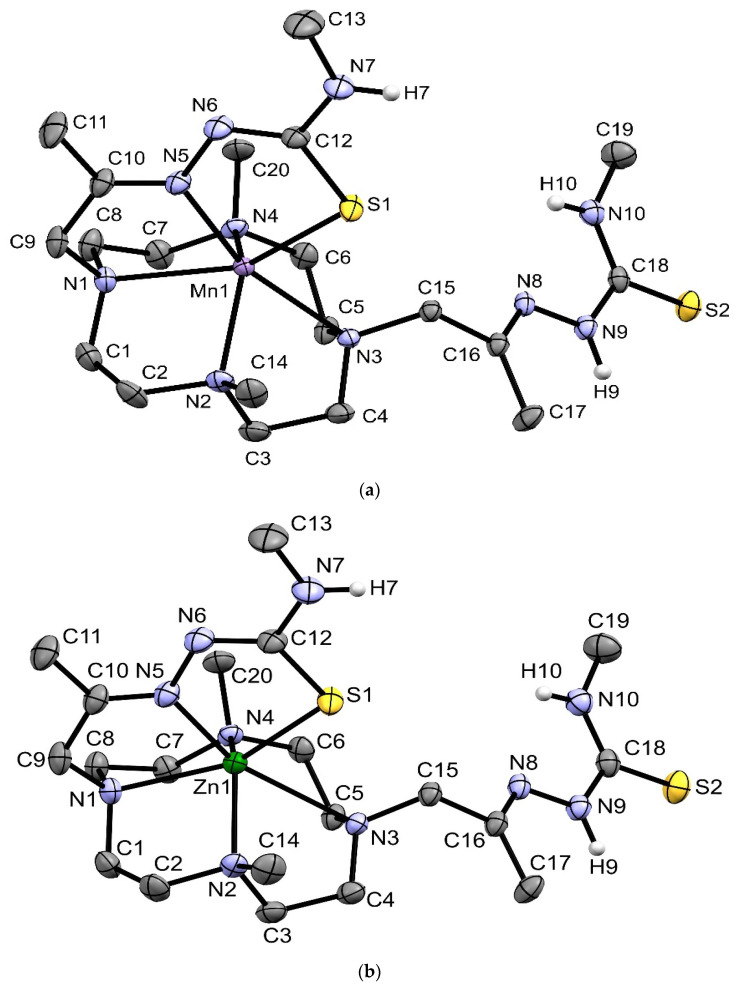
Single-crystal X-ray diffraction structure representations of the (**a**) [Mn1H**L**]^+^ and (**b**) [Zn1H**L**]^+^ complex cations at 30% thermal ellipsoid probability. Hydrogen atoms are omitted for clarity (except hydrogen atoms bound to nitrogen).

The coordination compounds [MnH**L**](BPh_4_), [CoH**L**](BPh_4_)·(C_3_H_6_O) and [ZnH**L**](BPh_4_)·1.67(C_3_H_6_O) crystallised in the monoclinic P2_1_/c space group. Each asymmetric unit contains two discrete diastereomeric complexes, [M1H**L**]^+^ and [M2H**L**]^+^, that are present as racemic mixtures of enantiomers in the unit cell. A single pendant thiosemicarbazonato arm is coordinated to the M^2+^ centre through the sulfur (S1) and azomethinic (N_azo_) nitrogen donor atoms (N5), along with the four macrocyclic (N_mac_) nitrogen atoms (N1, N2, N3 and N4), forming six five-membered chelate rings. The Mn^2+^, Co^2+^ and Zn^2+^ structures are consistent with the loss of one proton from the ligand. The C12–S1 bond lengths for the Mn^2+^ (1.745(2) Å), Co^2+^ (1.735(6) Å) and Zn^2+^ (1.743(3) Å) structures are indicative of more thiolate character than the thione character of the C18–S2 bond lengths (1.683(2) Å, 1.685(5) Å and 1.687(3) Å, respectively). Crystallographic data are shown in Table 1. Representations of the isostructural [Mn1H**L**]^+^ and [Zn1H**L**]^+^ complex cations are shown in Figure 2. A list of metal to donor bond lengths is given in Table 2. The donor atoms N1, N2, N3, N4, N5 and S1 are analogous to the donor atoms N11, N12, N13, N14, N15 and S3 of the Mn2, Co2 and Zn2 complexes.

The bonds between the metal ions and N3/N13 are significantly longer than the other bonds between the metal and nitrogen donor atoms of the macrocycle. This is presumably a result of the steric effects of the uncoordinated thiosemicarbazone pendant arm. The metal to sulfur donor bond lengths are typical of Mn^2+^ and Zn^2+^ thiosemicarbazonato complexes [11,20]. The Mn1, Mn2, Zn1, Zn2 and Co1 ions sit above the plane defined by the four nitrogen atoms of the macrocycle by 1.1 Å, while the Co2 ion sits above the plane by 1.0 Å.

**Figure 3:**
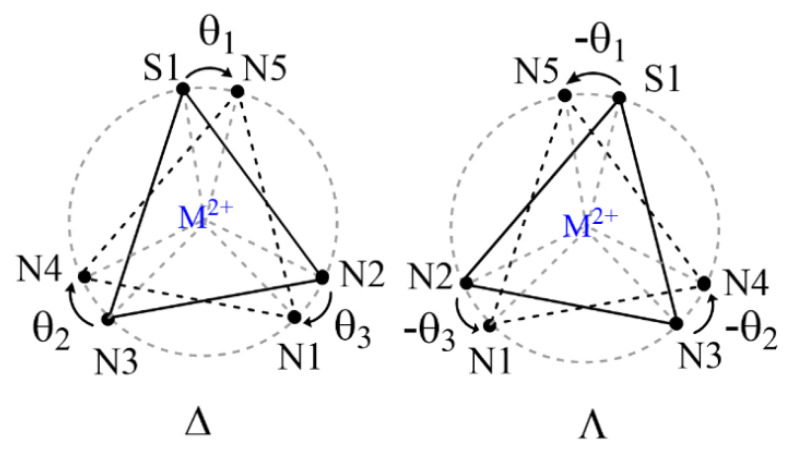
A representation of the torsion angles defined by the two trigonal planes.

The torsion angles defined by the N–C–C–N bonds in the macrocycle are described as δ (positive value) or λ (negative value) [69]. The average N–C–C–N torsion angles in the [Mn1H**L**]^+^ and [Mn2H**L**]^+^ complexes are ±58° and in [Zn1H**L**]^+^ and [Zn2H**L**]^+^ are ±57°. The trigonal distortion from an idealised octahedral geometry can be analysed using the dihedral angle (θ) defined as the twist angle between the corners of the trigonal planes [70]. The trigonal planes of the complexes are defined by N1-N4-N5 and N2-N3-S1 (Figure 3).

A positive torsion angle indicates the Δ enantiomer, and a negative torsion value indicates the Λ enantiomer. A trigonal prismatic geometry is indicated by a torsion angle of 0°, whereas a torsion angle of 60° indicates an octahedral geometry. The average torsion angles within the complexes are shown in Table 3. Interestingly, [Mn1H**L**]^+^ and [Zn1H**L**]^+^ exist as the Δ(δ,δ,δ,δ) and Λ(λ,λ,λ,λ) enantiomers, and [Mn2H**L**]^+^ and [Zn2H**L**]^+^ exist as the Δ(λ,λ,λ,λ) and Λ(δ,δ,δ,δ) enantiomers.

**Figure 4:**
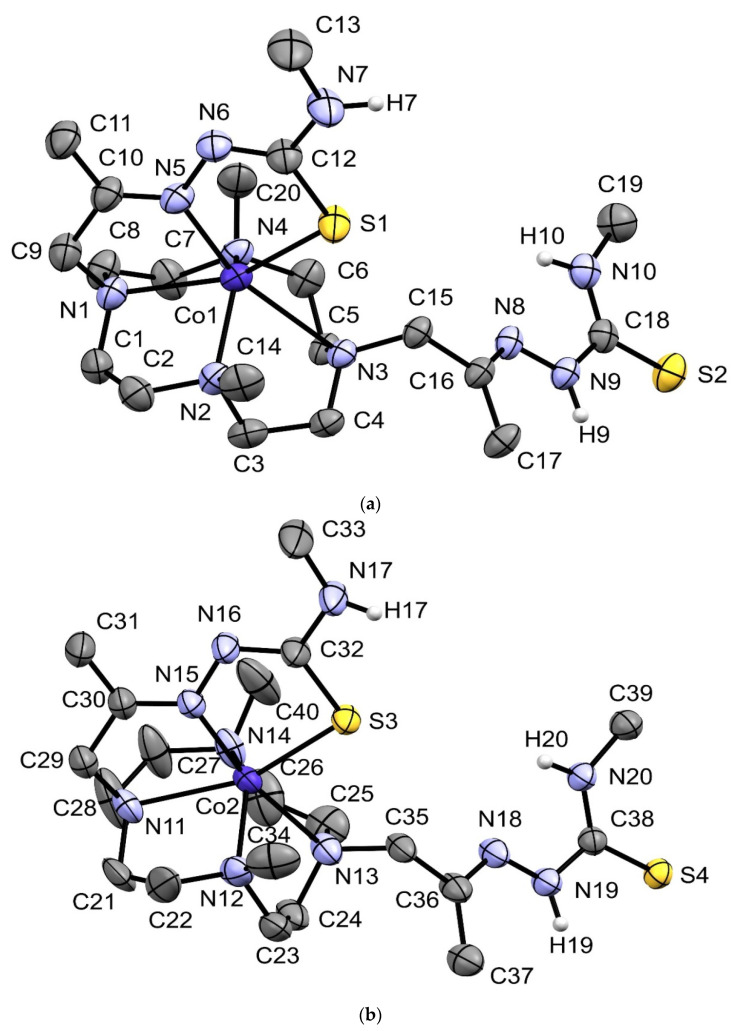
Single-crystal X-ray diffraction structure representations of the (**a**) [Co1H**L**]^+^ and (**b**) [Co2H**L**]^+^ complex cations at 30% thermal ellipsoid probability. Hydrogen atoms are omitted for clarity (except hydrogen atoms bound to nitrogen).

Representations of the [Co1H**L**]^+^ and [Co2H**L**]^+^ complex cations are shown in Figure 4. The [Co1H**L**]^+^ complex occurs as the Δ(δ,δ,δ,δ) and Λ(λ,λ,λ,λ) enantiomeric pair with average N–C–C–N torsion angles of ±55°. Interestingly, the [Co2H**L**]^+^ complex is a racemic mixture of the Δ(λ,λ,λ,δ) and Λ(δ,δ,δ,λ) enantiomeric pair. The N–C–C–N torsion angles in the Δ(λ,λ,λ,δ) enantiomer are 48.1°, 40.7°, 48.8° and −60.2°. This results in a Co2–N13 bond length of 2.317(4) Å, which is significantly shorter than the Co1–N3 bond length of 2.610(4) Å. The average dihedral angle for [Co2H**L**]^+^ is 30.9°, resulting in an intermediate geometry between trigonal prismatic and octahedral. The change in chirality probably reflects the competition between steric effects imposed by the ligand and the metal to donor atom bond length requirements of each of the complexes. Experimentally observed Co^2+^ complexes with (λ,λ,λ,δ) and (δ,δ,δ,λ) isomers have been reported previously in a complex with tetraethylcyclen and proline [71].

Several instances of trigonal prismatic high-spin 3d^5^ Mn^2+^, high-spin 3d^7^ Co^2+^ and 3d^10^ Zn^2+^ azamacrocyclic complexes have been reported [72,73,74,75,76,77]. While most six-coordinate complexes have a distinct octahedral preference over trigonal prismatic, high-spin d^5^ and d^10^ electron-configurations have no octahedral preference due to the absence of crystal field stabilisation energy, and high-spin d^7^ configurations have only a marginal preference [78]. A trigonal prismatic geometry can be forced upon Co^2+^ complexes by sterically constrained pendant arms, whereas two discrete ligands can give trigonal prismatic complexes with Mn^2+^ and Zn^2+^, including the formation of a Mn^2+^ complex with an N_4_S_2_ donor sphere [72,73,76,77]. Only two N_5_S cyclen-based complexes have been previously reported with Co^2+^ and Zn^2+^, both with octahedral geometry [79,80]. H_2_**L** is the first example of an azamacrocyclic N_5_S donor for Mn^2+^. A trigonal prismatic Zn^2+^ complex has been previously reported with a N_5_O ligand containing a single pendant arm [75].

### 2.4. Magnetic Susceptibility

The distorted coordination geometries of the [MnH**L**](BPh_4_) and [CoH**L**](BPh_4_) complexes suggested that the metal ions were high-spin 3d^5^ and 3d^7^, respectively. To confirm this hypothesis, the magnetic susceptibility of both the Mn^2+^ and Co^2+^ complexes were measured. The [MnH**L**](BPh_4_) complex had a magnetic moment of 5.82 B.M corresponding to five unpaired electrons (µ_s_ = 5.92 B.M.). The [CoH**L**](BPh_4_) complex had a magnetic moment of 4.24 B.M. corresponding to three unpaired electrons (µ_s_ = 3.87 B.M.). The difference from the spin-only value is due to the mixing of angular momentum from the excited state and spin-orbit coupling [81]. The Co^2+^ value indicates a spin quartet ground state, obtained from six-coordinate octahedral or trigonal prismatic geometry [82].

### 2.5. Density Functional Theory Calculations

Complexes incorporating the cyclen scaffold with pendant groups are known to have multiple stereoisomeric forms that result from the combination of the two chiral elements [83]. The diastereomers can have distinctly different coordination geometries and properties [77,84]. Furthermore, formation of a predominant diastereoisomer can result when there is a free-energy difference between the diastereomers [85,86]. Density functional theory (DFT) calculations were used to investigate the energetics of each species identified from the X-ray crystallography. The calculations were performed using the Becke, 3-parameter, Lee–Yang–Parr (B3LYP) functional for all complexes investigated. The standard Ahlrichs valence triple-ξ including polarization functions (TZVP) basis set was used for the high-spin Co^2+^ complexes and the DGDZVP basis set for the Zn^2+^ complexes. These combinations of functional and basis sets were chosen because they have shown good agreement with experimental values for similar combinations of ligands and metals [87,88]. For comparison with the XRD data, values for selected bond lengths of the optimised structures are shown in Table 4. The optimised geometries of [Co1H**L**]^+^, [Co2H**L**]^+^, [Zn1H**L**]^+^ and [Zn2H**L**]^+^ are provided in Figures S16–S23. A bond length difference of ~0.074 Å for Zn-S and ~0.078 Å for Zn-N_mac_ and Zn-N_azo_ was observed between the DFT optimised and XRD experimental values that was attributed to solvent effects.

DFT analysis of the [ZnH**L**]^+^ complexes in the presence of water indicated four energy minima corresponding to the following diastereomeric pairs: Δ(δ,δ,δ,δ) and Λ(λ,λ,λ,λ) for [Zn1H**L**]^+^, and Δ(λ,λ,λ,λ) and Λ(δ,δ,δ,δ) for [Zn2H**L**]^+^. According to these calculations, the minimum energy conformation corresponds to the Λ(λ,λ,λ,λ) isomer, with the relative energies of the Δ(δ,δ,δ,δ), Δ(λ,λ,λ,λ) and Λ(δ,δ,δ,δ) isomers being 0.34, 1.27 and 2.53 kJ mol^−1^, respectively. The optimisation of the Co^2+^ structures indicated that the minimum energy conformation corresponds to the Δ(δ,δ,δ,δ) isomer, with the relative energies of the Λ(λ,λ,λ,λ), Δ(λ,λ,λ,δ) and Λ(δ,δ,δ,λ) isomers being 4.17, 8.14 and 8.14 kJ mol^−1^, respectively.

### 2.6. Radiolabelling with ^68^Ga^3+^

**Figure 5:**
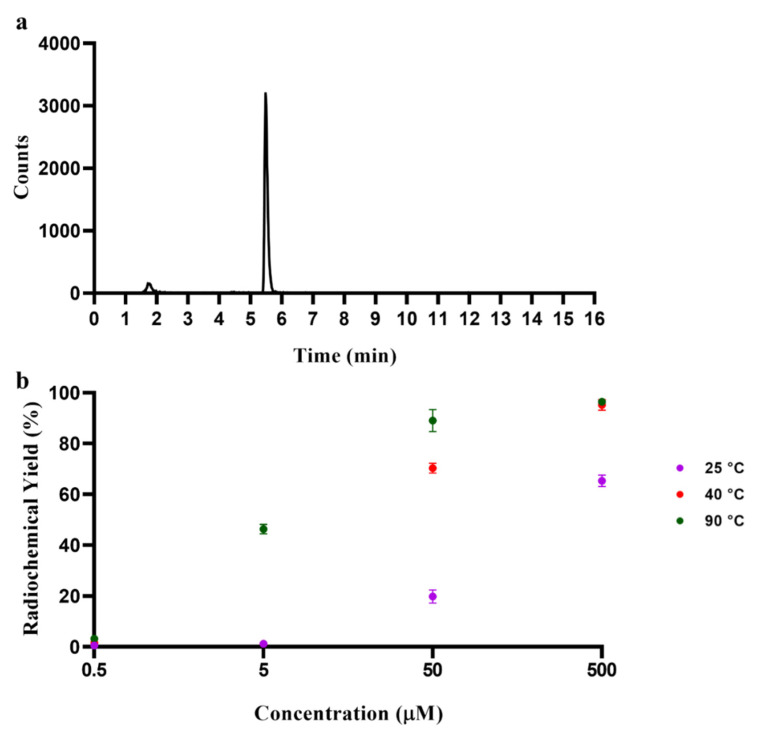
(**a**) Radio-HPLC chromatogram of the reaction between ^68^Ga^3+^ (1.2 MBq) and H_2_**L** (50 µM) after 10 min at pH 6 and 90 °C. (**b**) Radiochemical yields for the reaction of ^68^Ga^3+^ at various concentrations (0.5 to 500 μM) and temperatures (25, 40 and 90 °C) at pH 6 after 10 min.

Preliminary radiolabelling studies were performed with ^68^Ga^3+^ eluted from a ^68^Ge/^68^Ga generator. The eluate was buffered with 1 M sodium acetate to pH 3.5 or 6 and reacted with H_2_**L** (0.5, 5, 50 and 500 μM) at ambient temperature, 40 and 90 °C for 10 min. The 68 min half-life of ^68^Ga^3+^ necessitates relatively short reaction times. The reaction mixtures were analysed by radio-HPLC, which showed a single product with a retention time (*R*_T_) of 5.3 min while there was negligible retention of the [^68^Ga]Ga^3+^ ion on the C18 column (*R*_T_ = 1.5 min). The synthesis of the non-radioactive Ga^3+^ complex was attempted but isolation was unsuccessful. The radiolabelling reaction with DOTA was performed previously under the same conditions and used as a comparison [49]. The percentage of ^68^Ga^3+^ incorporation is dependent on ligand concentration, pH and temperature. ^68^Ga^3+^ incorporation was investigated at both pH 3.5 and pH 6 at 50 µM with an activity to ligand ratio of 0.2 MBq nmol^−1^ (Figure 5). A radiochemical yield (RCY) > 95% was achieved at pH 6 at both 40 and 90 °C but required a ligand concentration of 500 µM (Table 5). At pH 3.5, a RCY > 95% was achieved with a ligand concentration of 50 µM at 90 °C.

Under similar conditions, DOTA required concentrations of 50 µM or above to reach yields > 95%, similar to H_2_**L**. At pH 6, a RCY > 95% for DOTA was achieved with a concentration of 500 μM at 25 °C or 50 μM at 90 °C [49]. The RCY for DOTA with a concentration of 50 μM at 25 °C is ~87% whereas H_2_**L** achieved only 2% RCY under the same conditions. These results demonstrate that H_2_**L** radiolabelling with ^68^Ga^3+^ is temperature dependent at both pH values studied, and that it does not possess the radiolabelling efficiency properties to be a potential alternative chelator for ^68^Ga^3+^ radiolabelling. This is perhaps unsurprising given the combination of soft base sulfur donor atoms and the hard acid Ga^3+^ [89]. A ligand design incorporating oxygen donor-containing semicarbazone pendant arms may be better suited for use in ^68^Ga^3+^ radiopharmaceuticals [90].

## 3. Materials and Methods

### 3.1. General Procedures

Reagents were purchased from standard commercial sources unless otherwise stated and used without further purification. Cyclen was purchased from Strem Chemicals (Newburyport, MA, USA). Nuclear magnetic resonance (NMR) data were collected on a Bruker AVANCE III 600 (^1^H at 600.27 MHz, ^13^C{^1^H} at 150.95 MHz) (Bruker, Billerica, MA, USA). Spectra were processed using MestReNova 10.0 software. DMSO-*d*_6_ was obtained from Cambridge Isotope Laboratories Inc. Chemical shifts (δ) are reported in parts per million (ppm) with respect to TMS and are referenced to residual solvent peaks. Coupling constants (*J*) are reported in Hz. Unless specified, all NMR spectra were recorded at 25 °C. Low-resolution mass spectrometry (LR–MS) was carried out using an Agilent 1260 Infinity liquid chromatograph system coupled with a 6120 series quadrupole mass spectrometer (Agilent Technologies, Santa Clara, CA, USA) in MeOH using ESI. High-resolution mass spectrometry (HR–MS) was carried out with an Agilent 6540 UHD Accurate Mass Q-TOF LCMS (Agilent Technologies, Santa Clara, CA, USA). The mass spectrometer was fitted with the Agilent Jet Stream Source using ESI. Positive detection is shown by the charge on the ion, e.g., [M + H]^+^ for a positive protonated ion. All calculated values were determined using the PerkinElmer software ChemDraw^®^ Professional 19.0 to four decimal places. HPLC traces of both radiolabelled and non-radiolabelled complexes were acquired using a Shimadzu HPLC system (Shimadzu, Kyoto, Japan) with a Phenomenex Luna C18(2) column (4.6 mm × 150 mm, 5 μm), a 1 mL/min flow rate gradient elution of 0.05% TFA in 5% MeCN in H_2_O to 100% MeCN over 15 min at 25 °C with UV spectroscopic detection at 254 nm and 280 nm. Data were processed and analysed using Laura radio chromatography software (Lablogic, Brandon, FL, USA). Magnetic susceptibility was measured at room temperature by calibrating a Johnson Matthey MSB balance with [Ni(en)_3_]S_2_O_3_ at 295 K and diamagnetic corrections of the paramagnetic susceptibilities were calculated using standard Pascal’s constants [91,92].

### 3.2. Synthesis

1,1′-(4,10-Dimethyl-1,4,7,10-tetraazacyclododecane-1,7-diyl)bis(propan-2-one) (**1**) was prepared according to previously published methods [63].

H_2_**L**: To a solution of **1** (1.432 g, 4.582 mmol, 1 equiv.) and 4-methyl-3-thiosemicarbazide (1.060 g, 10.08 mmol, 2.2 equiv.) in EtOH (40 mL) was added 7 drops of glacial acetic acid. The solution was stirred for 72 h at room temperature. The precipitate was collected on a vacuum glass frit, washed with EtOAc and Et_2_O to afford a light-yellow solid (0.6532 g, 1.342 mmol, 29%). RP-HPLC: R_T_ = 5.27 min; ^1^H NMR (600 MHz, DMSO-d_6_): δ = 1.96 (s, 6H, CH_2_CCH_3_), 2.45 (s, 6H, CH_2_NCH_3_), 2.75 (s, 16H, NCH_2_CH_2_N), 2.98 (d, 6H, CSNCH_3_, ^3^*J*_H-H_ = 4.6 Hz), 3.34 (s, 4H, NCH_2_C), 8.18 (d, 2H, CSNHCH_3_ ^3^*J*_H-H_ = 4.6 Hz), 10.03 (s, 2H, NNHCS); ^13^C{^1^H} NMR (150 MHz, DMSO-d_6_): δ = 15.8 (CH_2_CCH_3_), 30.9 (CSNCH_3_), 40.1 (CH_2_NCH_3_), 49.8 (NCH_2_CH_2_N), 63.3 (NCH_2_C), 150.8 (C=N), 178.8 (C=S); HRMS (ESI) [H_2_**L** + H]^+^: *m/z* = 487.3199 (experimental), 487.3035 (calculated).

#### General Procedure for the Synthesis of the Complexes

To a solution of H_2_**L** (0.0500 g, 0.103 mmol, 1 equiv.) in MeOH (4 mL) was added a solution of Mn(OAc)_2_·4H_2_O, Co(OAc)_2_·4H_2_O or Zn(OAc)_2_·2H_2_O (0.103 mmol, 1 equiv.) in MeOH (2 mL), and the solution was left to stir for 17 h at ambient temperature. A solution of NaBPh_4_ (0.0352 g, 0.103 mmol, 1 equiv.) in MeOH (3 mL) was added and the resulting suspension was stirred for an additional 4 h at ambient temperature. The product was collected on a glass frit and washed with Et_2_O. ([MnH**L**](BPh_4_): 0.0498 g, 0.0579 mmol, 56%), ([CoH**L**](BPh_4_): 0.0271 g, 0.0314 mmol, 30%), ([ZnH**L**](BPh_4_): 0.0565 g, 0.0649 mmol, 63%). The solids were each dissolved in acetone (1 mL) and Et_2_O was vapour diffused to form crystals suitable for single-crystal X-ray diffraction. RP-HPLC [MnH**L**][BPh_4_]: R_T_ = 6.06 min; [CoH**L**][BPh_4_]: R_T_ = 6.05 min; [ZnH**L**][BPh_4_]: R_T_ = 6.01 min; ^1^H NMR [ZnH**L**](BPh_4_) (600 MHz, DMSO-d_6_): δ = 1.96 (s, 3H, CH_3_), 2.03 (s, 3H, CH_3_), 2.31 (s, 6H, CH_3_), 2.40, 2.59, 2.72, 2.88, 3.04 (m, 16H, NCH_2_CH_2_N), 2.74 (d, 3H, CH_3_, ^3^*J*_H-H_ = 4.3 Hz), 2.98 (d, 3H, CH_3_, ^3^*J*_H-H_ = 4.5 Hz), 3.40 (s, 2H, NCH_2_CN), 3.60 (s, 2H, NCH_2_CN), 6.78 (t, 4H, ArH, ^3^*J*_H-H_ = 7.0 Hz), 6.92 (t, 8H, ArH, ^3^*J*_H-H_ = 7.5 Hz), 7.17 (m, 8H, ArH), 6.56 (q, 1H, NHCH_3_), 8.22 (q 1H, NHCH_3_), 10.01 (s, 2H, NH); ^13^C{^1^H NMR (150 MHz, DMSO-d_6_): δ = 17.2 (CH_2_CCH_3_), 18.1 (CH_2_CCH_3_), 28.9 (CSNCH_3_), 30.7 (CSNCH_3_), 45.2 (CH_2_NCH_3_), 51.8, 54.1, 54.9 (NCH_2_CH_2_N), 58.7 (NCH_2_CN), 61.3 (NCH_2_CN), 121.5 (ArC), 125.3 (ArC), 135.5 (ArC), 148.6 (C=N), 157.3 (C=N), 163.2 (ArCB), 179.8 (C=S); HRMS (ESI) [ZnH**L** + H]^+^: *m/z* = 549.2249 (experimental), 549.2249 (calculated); [MnH**L** + H]^+^: *m/z* = 540.2335 (experimental), 540.2338 (calculated); [CoH**L** + H]^+^: *m/z* = 544.2298 (experimental), 544.2289 (calculated).

### 3.3. Radiolabelling with ^68^Ga

^68^Ga was eluted from an Eckert and Ziegler ^68^Ge/^68^Ga generator system (Eckert and Ziegler, Berlin, Germany). Aqueous HCl solution (0.1 M, 5 mL) was passed through the generator and the eluate was collected in five 1 mL fractions. Aqueous NaOAc (1 M) was added to the fourth fraction (1 mL, containing ~77.6 MBq ^68^Ga) to increase the pH to either pH 3.5 or pH 6. Aliquots from the pH adjusted fraction were used for radiolabelling reactions. H_2_**L** was dissolved in DMSO (1 mg/mL) and diluted with ultrapure water. ^68^Ga (15 µL, ~1.16 MBq in pH adjusted solution) was added to chelator solutions (105 µL) to provide solutions with chelator concentrations ranging from 0.5–500 µM and the final reaction solution was incubated at 25 °C, 40 °C or 90 °C for 10 min. The reaction solutions were analysed via radio-HPLC (5–20 µL injection).

### 3.4. Single-Crystal X-ray Diffraction Procedure

Low-temperature (123 K or 173 K) X-ray intensity data were collected using a Rigaku XtaLAB Synergy diffractometer (Rigaku Oxford Diffraction, Chalgrove, Oxford, United Kingdom) fitted with a Hypix6000HE hybrid photon counting detector and MoKα (*λ* = 0.71073 Å) or CuKα (*λ* = 1.54184 Å) radiation. Data were processed, including a multiscan absorption correction, using the proprietary diffractometer software package CrysAlisPro v1.171.39.46 [93]. The structure was solved by conventional methods and refined on *F*^2^ using full matrix least squares using the SHELX 2018/3 software suite [94]. Non-hydrogen atoms were refined with anisotropic displacement parameters. Hydrogen atoms attached to carbon were placed in calculated positions and were refined using a riding model. The positions of the acidic hydrogen atoms attached to nitrogen were initially located in the difference Fourier map but were included in calculated positions and refined using a riding model. In the [CoH**L**](BPh_4_) and [ZnH**L**](BPh_4_) complexes, solvent molecules were successfully modelled. However, after refinement of the primary molecule [MnH**L**](BPh_4_), residual electron density was assumed to be isolated solvent molecules located in the crystal lattice. These were accounted for using PLATON/SQUEEZE [95]. CSD reference numbers 2072659-2072661.

### 3.5. DFT Calculations

Density functional theory (DFT) calculations were performed using the Gaussian 16 program package with the Becke, 3-parameter, Lee–Yang–Parr (B3LYP) functional and the DGDZVP basis set for the Zn^2+^ complexes [96,97,98,99,100]. The B3LYP functional and the standard Ahlrichs valence triple-ξ including polarization functions (TZVP) basis set were used for the high-spin Co^2+^ complexes [101,102]. The geometries of the various complexes were fully optimised without imposing any symmetry constraint. No imaginary frequencies were found at the optimised molecular geometries, which indicate that they are real minima of the potential energy surface. The complexes were optimised in aqueous solution by using the polarizable continuum model with the integral equation formalism variant (IEFPCM), which creates a solvent cavity via a set of overlapping spheres [103]. The calculated relative energies of the complexes include nonpotential energy contributions. Calculation results were visualized and interpreted using GaussView version 6.1.1 [104].

## 4. Conclusions

Hybrid thiosemicarbazone ligands are of interest in the development of biologically-active metal complexes and radiopharmaceuticals. A variant of cyclen with two thiosemicarbazone pendant groups was synthesised and the coordination complexes with Mn^2+^, Co^2+^ and Zn^2+^ were prepared and characterised. ^1^H NMR spectroscopy of [ZnH**L**](BPh_4_) indicated the formation of a singly deprotonated ligand with one pendant arm coordinated to the metal centre. X-ray crystallography of [MnH**L**](BPh_4_) and [ZnH**L**](BPh_4_) indicated the formation of the Δ(δ,δ,δ,δ)/Λ(λ,λ,λ,λ) and Δ(λ,λ,λ,λ)/Λ(δ,δ,δ,δ) diastereomers, whereas [CoH**L**](BPh_4_) was present as the Δ(δ,δ,δ,δ)/Λ(λ,λ,λ,λ) and Δ(λ,λ,λ,δ)/Λ(δ,δ,δ,λ) diastereomers. The distorted trigonal prismatic geometries of the high-spin Mn^2+^, Co^2+^ and Zn^2+^ complexes are a result of the steric constraints of the ligand. DFT calculations indicated small differences in the relative energies of the diastereomers, which suggests that they likely also exist in solution. Further studies are required to indicate whether the particular diastereomers could have distinct biological activities. Radiolabelling with ^68^Ga^3+^ produced a single species at ligand concentrations between 50 and 500 µM at 90 °C to achieve RCY > 95%, indicating the potential of hybrid thiosemicarbazone ligands with radiometals in the development of radiopharmaceuticals. Investigations into additional metal ions and their properties are in progress.

## Figures and Tables

**Table 1:** Experimental, crystallographic and refinement data for [MnH**L**](BPh_4_), [CoH**L**](BPh_4_)·(C_3_H_6_O) and [ZnH**L**](BPh_4_)·1.67(C_3_H_6_O).

	[MnHL](BPh_4_)	[CoHL](BPh_4_)·(C_3_H_6_O)	[ZnHL](BPh_4_)·1.67(C_3_H_6_O)
**Empirical formula**	C_44_H_61_BMnN_10_S_2_	C_47_H_67_BCoN_10_OS_2_	C_49_H_70.75_BN_10_O_1.67_S_2_Zn
**Formula weight**	859.89	921.96	966.86
**Crystal system**	Monoclinic	Monoclinic	Monoclinic
**Space group**	P21/c	P21/c	P21/c
**Temperature (K)**	123(2)	173(2)	123(1)
**a (Å)**	17.4842(3)	17.2017(2)	17.32220(10)
**b (Å)**	35.9904(5)	35.8919(4)	36.0585(2)
**c (Å)**	17.4662(3)	17.3716(2)	17.36950(10)
**α (°)**	90	90	90
**β (°)**	100.8860(10)	102.2170(10)	101.7190(10)
**γ (°)**	90	90	90
**V (Å^3^)**	10,793.1(3)	10,482.4	10,623.06(11)
**Z**	8	8	8
**Dc (g cm^−3^)**	1.058	1.168	1.209
**Absorption coefficient (mm^−1^)**	0.358	3.639	1.729
**F(000)**	3656	3928	4121
**Angle range 2θ, °**	MoKα 6.630 to 55.754	CuKα 7.168 to 154.048	CuKα 7.012 to 154.846
**Reflections collected**	143,110	102,026	115,251
**Independent reflections**	25,679 [*R*(int) = 0.0469]	21,504 [*R*(int) = 0.0832]	22,186 [*R*(int) = 0.0456]
**Final *R*_1_ values (*I* > 2** **σ** **(*I*))**	0.0440	0.0889	0.0572
**Final *wR*_1_(*F*^2^) values (*I* > 2σ(*I*))**	0.1111	0.2389	0.1622
**Final *R*_1_ values (all data)**	0.0606	0.1252	0.0617
**Final *wR*_1_(*F*^2^) values (all data)**	0.1180	0.2764	0.1667
**GoF on F^2^**	1.076	1.036	1.023
**CSD no.**	2,072,659	2,072,660	2,072,661

**Table 2:** Metal to donor bond lengths for [Mn1H**L**]^+^, [Mn2H**L**]^+^, [Co1H**L**]^+^, [Co2H**L**]^+^, [Zn1H**L**]^+^ and [Zn2H**L**]^+^.

	Mn1 (Å)	Mn2 (Å)	Co1 (Å)	Co2 (Å)	Zn1 (Å)	Zn2 (Å)
**M-N1/11**	2.362(2)	2.397(1)	2.331(4)	2.325(4)	2.340(2)	2.393(2)
**M-N2/12**	2.264(2)	2.254(2)	2.156(4)	2.183(5)	2.152(2)	2.140(2)
**M-N3/13**	2.501(2)	2.471(2)	2.620(4)	2.317(4)	2.737(2)	2.630(2)
**M-N4/14**	2.244(2)	2.250(2)	2.113(4)	2.159(4)	2.121(2)	2.125(2)
**M-N5/15**	2.257(2)	2.218(2)	2.120(4)	2.087(4)	2.147(2)	2.130(2)
**M-S1/3**	2.462(1)	2.455(1)	2.323(2)	2.365(1)	2.346(7)	2.344(7)

**Table 3:** The dihedral angles between the two trigonal planes in the [Mn1H**L**]^+^, [Mn2H**L**]^+^, [Co1H**L**]^+^, [Co2H**L**]^+^, [Zn1H**L**]^+^ and [Zn2H**L**]^+^ metal centres.

	θ_1_ (°)	θ_2_ (°)	θ_3_ (°)	Av (°)
**Mn1**	35.7	20.2	8.1	21.4
**Mn2**	32.0	18.6	7.0	19.2
**Co1**	37.5	21.4	12.3	23.7
**Co2**	40.7	31.2	20.9	30.9
**Zn1**	37.4	21.7	13.1	24.1
**Zn2**	33.7	21.5	12.6	22.6

**Table 4:** Selected bond lengths (Å) of the optimised and XRD structures of [Co1H**L**]^+^, [Co2H**L**]^+^, [Zn1H**L**]^+^ and [Zn2H**L**]^+^.

	Zn1 XRD	Zn1 DFT	Zn2 XRD	Zn2 DFT	Co1 XRD	Co1 DFT	Co2 XRD	Co2 DFT
**M-N1/N11**	2.340(2)	2.390	2.393(2)	2.411	2.331(4)	2.351	2.325(4)	2.402
**M-N2/N12**	2.151(2)	2.240	2.140(2)	2.260	2.156(4)	2.248	2.183(5)	2.259
**M-N3/N13**	2.737(2)	2.727	2.630(2)	2.701	2.620(4)	2.680	2.317(4)	2.400
**M-N4/N14**	2.120(2)	2.228	2.125(2)	2.238	2.113(4)	2.217	2.159(4)	2.239
**M-N5N/15**	2.147(2)	2.211	2.130(2)	2.212	2.120(4)	2.164	2.087(4)	2.128
**M-S1/S3**	2.346(7)	2.420	2.344(7)	2.419	2.323(2)	2.401	2.365(1)	2.412

**Table 5:** The radiochemical yields for the reaction of ^68^Ga^3+^ with H_2_**L** (50 µM) and DOTA (50 µM) at pH 3.5 and pH 6 and various temperatures (25, 40 and 90 °C) [49].

	H_2_L		DOTA	
	pH 3.5	pH 6	pH 3.5	pH 6
**25 °C**	1.8 ± 1.7	19.8 ± 2.6	86.7 ± 5.0	73.2 ± 6.4
**40 °C**	60.4 ± 3.1	70.3 ± 1.9	-	-
**90 °C**	95.1 ± 2.3	89.0 ± 4.3	95.3 ± 0.9	97.2 ± 0.3

## Data Availability

Data are available within the article or Supplementary Information.

## Supplementary Materials

The following are available online: ^1^H and ^13^C{^1^H} NMR spectra, ESI–MS spectra, HPLC spectra and computational methods.

## Sample Availability

Samples of compounds H_2_**L**, [MnH**L**](BPh_4_), [CoH**L**](BPh_4_) and [ZnH**L**](BPh_4_) are available from the authors.
