# Supplementary material for: The Synthesis of a Bis(thiosemicarbazone) Macrocyclic Ligand and the Mn(II), Co(II), Zn(II) and 68Ga(III) Complexes
Source: Molecules. 2021 Jun 15;26(12):3646. doi: 10.3390/molecules26123646 (PMC8232287; doi:10.3390/molecules26123646)
Supplement: Supplementary file 1 [file molecules-26-03646-s001.zip › molecules-1249066-SI.pdf]

The Synthesis of a Bis(thiosemicarbazone) Macrocyclic Ligand and the Mn(II), Co(II), Zn(II) and ^{68}Ga(III) Complexes

Supporting Information

Melyssa L. Grieve,[a] Patrick R. W. J. Davey,[a] Craig M. Forsyth,[a] and Brett M. Paterson[a,b]*

[a] School of Chemistry, Monash University, Clayton, Victoria 3800, Australia

[b] Monash Biomedical Imaging, Monash University, Clayton, Victoria 3800, Australia

* Corresponding author: Brett Paterson (brett.paterson@monash.edu)

Table of Contents

NMR Spectra of Compounds..S5

Figure S1 The ^{1}H NMR spectrum of $H_2\mathbf{L^1}$ in d_6-DMSO (top) and the ^{13}C NMR spectrum of $H_2\mathbf{L^1}$ in d_6-DMSO (bottom). Residual solvent peaks are marked with an asterisk...S5

Figure S2 The ^{1}H-^{1}H COSY NMR spectrum of $H_2\mathbf{L}$..S6

Figure S3 The variable-temperature ^{1}H NMR spectrum of $H_2\mathbf{L}$. The lowest temperature (shown in pink) is at 25 °C, followed by 30, 40, 50, 60, 70 and 80 °C (shown in red)...S6

Figure S4 The ^{1}H NMR spectrum of [ZnH**L**][BPh_4] in d-DMSO...S7

Figure S5 The $^{13}C\{^{1}H\}$ NMR spectrum of [ZnH**L**][BPh_4] in d-DMSO..S7

Figure S6 The variable-temperature ^{1}H NMR spectrum of [ZnH**L**][BPh_4...S8

Mass Spectrometry Data...S8

Figure S7 The high-resolution mass spectrum of $H_2\mathbf{L}$...S8

Figure S8 The high-resolution mass spectrum of $[MnH\mathbf{L}]^+$..S9

Figure S9 The high-resolution mass spectrum of $[CoH\mathbf{L}]^+$…..S9

Figure S10 The high-resolution mass spectrum of $[ZnH\mathbf{L}]^+$..S9

Analytical RP-HPLC Traces..S10

Figure S11 The analytical RP-HPLC trace of $H_2\mathbf{L}$...S10

Figure S12 The analytical RP-HPLC trace of $[MnH\mathbf{L}]^+$..S10

Figure S13 The analytical RP-HPLC trace of $[CoH\mathbf{L}]^+$..S11

Figure S14 The analytical RP-HPLC trace of $[ZnH\mathbf{L}]^+$..S11

Radiochemistry Data...S12

Figure S15 The RCY of [^{68}Ga][GaH$\mathbf{L^1}$] at pH 3.5 and pH 6 at 25, 40 and 90 °C................................S12

Table S1 The radiochemical yields for the reaction of ^{68}Ga with $H_2\mathbf{L}$ (0.5, 5, 50 and 500 µM) at pH 3.5 and pH 6 and different temperatures (25, 40 and 90 °C)..S12

DFT Structures and Data..S13

Figure S16 Optimised structure of Co^{2+} Δ(δ,δ,δ,δ) isomer at the B3LYP/TZVP level of theory...............S13

Table S2 Selected calculated bond lengths (Å) for the Co^{2+} Δ(δ,δ,δ,δ) isomer at the B3LYP/TZVP level of theory..S13

Table S3 Selected calculated bond angles (˚) for the Co^{2+} Δ(δ,δ,δ,δ) isomer at the B3LYP/TZVP level of theory..S13

Table S4 Calculated torsion angles (˚) for the Co^{2+} Δ(δ,δ,δ,δ) isomer at the B3LYP/TZVP level of theory..S14

Figure S17 Optimised structure of Co^{2+} Λ(λ,λ,λ,λ) isomer at the B3LYP/TZVP level of theory..............S14

Table S5 Selected calculated bond lengths (Å) for the Co^{2+} Λ(λ,λ,λ,λ) isomer at the B3LYP/TZVP level of theory..........S14

Table S6 Selected calculated bond angles (˚) for the Co^{2+} Λ(λ,λ,λ,λ) isomer at the B3LYP/TZVP level of theory..........S15

Table S7 Calculated torsion angles (˚) for the Co^{2+} Λ(λ,λ,λ,λ) isomer at the B3LYP/TZVP level of theory..........S15

Figure S18 Optimised structure of Co^{2+} Δ(λ,λ,λ,δ) isomer at the B3LYP/TZVP level of theory..............S15

Table S8 Selected calculated bond lengths (Å) for the Co^{2+} Δ(λ,λ,λ,δ) isomer at the B3LYP/TZVP level of theory..........S16

Table S9 Selected calculated bond angles (˚) for the Co^{2+} Δ(λ,λ,λ,δ) isomer at the B3LYP/TZVP level of theory..........S16

Table S10 Calculated torsion angles (˚) for the Co^{2+} Δ(λ,λ,λ,δ) isomer at the B3LYP/TZVP level of theory..........S16

Figure S19 Optimised structure of Co^{2+} Λ(δ,δ,δ,λ) isomer at the B3LYP/TZVP level of theory..............S17

Table S11 Selected calculated bond lengths (Å) for the Co^{2+} Λ(δ,δ,δ,λ) isomer at the B3LYP/TZVP level of theory..........S17

Table S12 Selected calculated bond angles (˚) for the Co^{2+} Λ(δ,δ,δ,λ) isomer at the B3LYP/TZVP level of theory..........S17

Table S13 Calculated torsion angles (˚) for the Co^{2+} Λ(δ,δ,δ,λ) isomer at the B3LYP/TZVP level of theory..........S18

Figure S20 Optimised structure of Zn^{2+} Δ(δ,δ,δ,δ) isomer at the B3LYP/DGDZVP level of theory.........S18

Table S14 Selected calculated bond lengths (Å) for the Zn^{2+} Δ(δ,δ,δ,δ) isomer at the B3LYP/TZVP level of theory..........S18

Table S15 Selected calculated bond angles (˚) for the Zn^{2+} Δ(δ,δ,δ,δ) isomer at the B3LYP/TZVP level of theory..........S19

Table S16 Calculated torsion angles (˚) for the Zn^{2+} Δ(δ,δ,δ,δ) isomer at the B3LYP/TZVP level of theory..........S19

Figure S21 Optimised structure of Zn^{2+} Λ(λ,λ,λ,λ) isomer at the B3LYP/DGDZVP level of theory.........S19

Table S17 Selected calculated bond lengths (Å) for the Zn^{2+} Λ(λ,λ,λ,λ) isomer at the B3LYP/TZVP level of theory..........S20

Table S18 Selected calculated bond angles (˚) for the Zn^{2+} Λ(λ,λ,λ,λ) isomer at the B3LYP/TZVP level of theory..........S20

Table S19 Calculated torsion angles (˚) for the Zn^{2+} Λ(λ,λ,λ,λ) isomer at the B3LYP/TZVP level of theory..........S20

Figure S22 Optimised structure of Zn^{2+} Δ(λ,λ,λ,λ) isomer at the B3LYP/DGDZVP level of theory.........S21

Table S20 Selected calculated bond lengths (Å*)* for the Zn^{2+} Δ(λ,λ,λ,λ) isomer at the B3LYP/TZVP level of theory....S21

Table S21 Selected calculated bond angles (˚) for the Zn^{2+} Δ(λ,λ,λ,λ) isomer at the B3LYP/TZVP level of theory....S21

Table S22 Calculated torsion angles (˚) for the Zn^{2+} Δ(λ,λ,λ,λ) isomer at the B3LYP/TZVP level of theory....S22

Figure S23 Optimised structure of Zn^{2+} Λ(δ,δ,δ,δ) isomer at the B3LYP/DGDZVP level of theory.........S22

Table S23 Selected calculated bond lengths (Å*)* for the Zn^{2+} Λ(δ,δ,δ,δ) isomer at the B3LYP/TZVP level of theory....S22

Table S24 Selected calculated bond angles (˚) for the Zn^{2+} Λ(δ,δ,δ,δ) isomer at the B3LYP/TZVP level of theory....S23

Table S25 Calculated torsion angles (˚) for the Zn^{2+} Λ(δ,δ,δ,δ) isomer at the B3LYP/TZVP level of theory....S23

Table S26 Cartesian coordinates for the Co^{2+} Δ(δ,δ,δ,δ) isomer....S24

Table S27 Cartesian coordinates for the Co^{2+} Λ(λ,λ,λ,λ) isomer....S25

Table S28 Cartesian coordinates for the Co^{2+} Δ(λ,λ,λ,δ) isomer....S27

Table S29 Cartesian coordinates for the Co^{2+} Λ(δ,δ,δ,λ) isomer....S29

Table S30 Cartesian coordinates for the Zn^{2+} Λ(δ,δ,δ,δ) isomer....S30

Table S31 Cartesian coordinates for the Zn^{2+} Δ(λ,λ,λ,λ) isomer....S32

Table S32 Cartesian coordinates for the Zn^{2+} Δ(δ,δ,δ,δ) isomer....S34

Table S33 Cartesian coordinates for the Zn^{2+} Λ(λ,λ,λ,λ) isomer....S36

NMR Spectra of Compounds

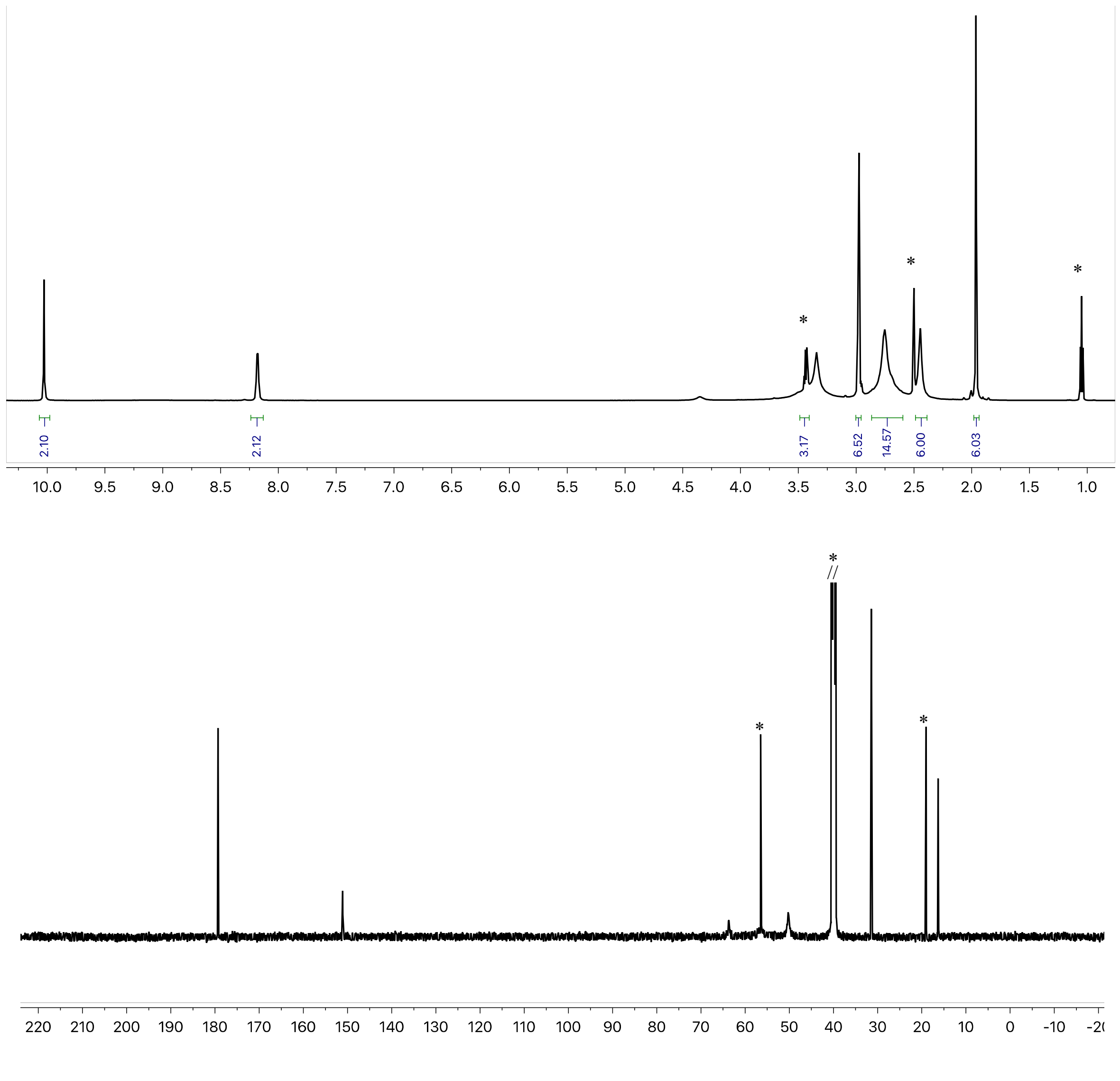

Figure S1. The ^{1}H NMR spectrum of $H_2\mathbf{L^1}$ in d_6-DMSO (top) and the ^{13}C NMR spectrum of $H_2\mathbf{L^1}$ in d_6-DMSO (bottom). Residual solvent peaks are marked with an asterisk.

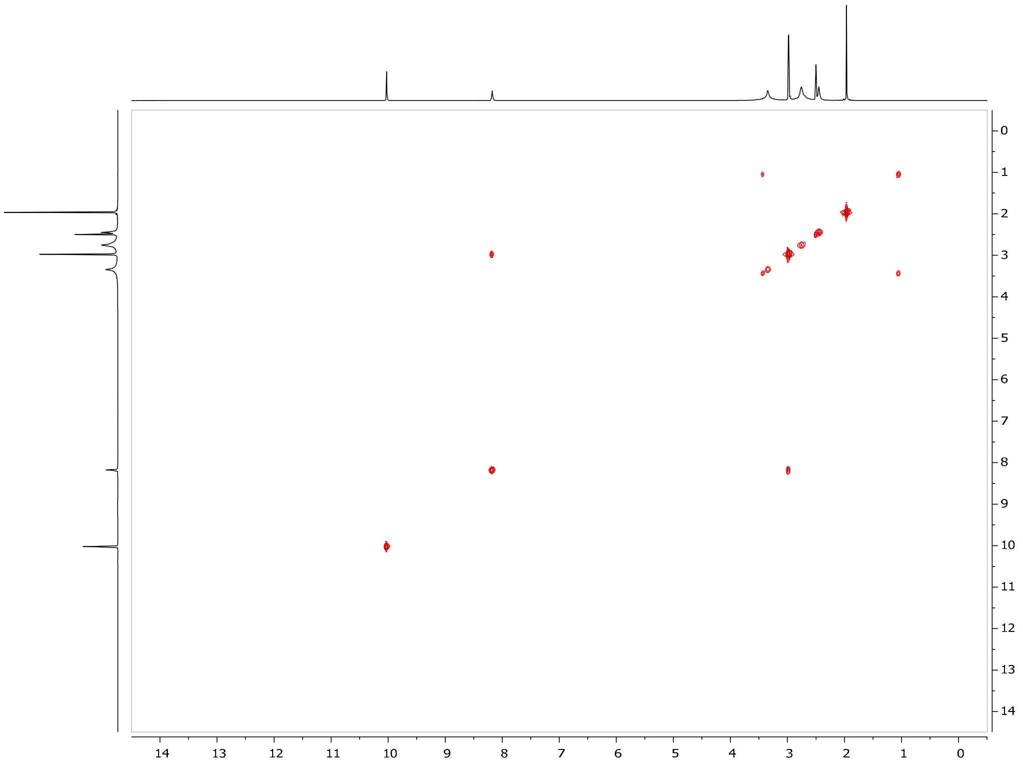

Figure S2. The ^{1}H-^{1}H COSY NMR spectrum of $H_2\mathbf{L}$.

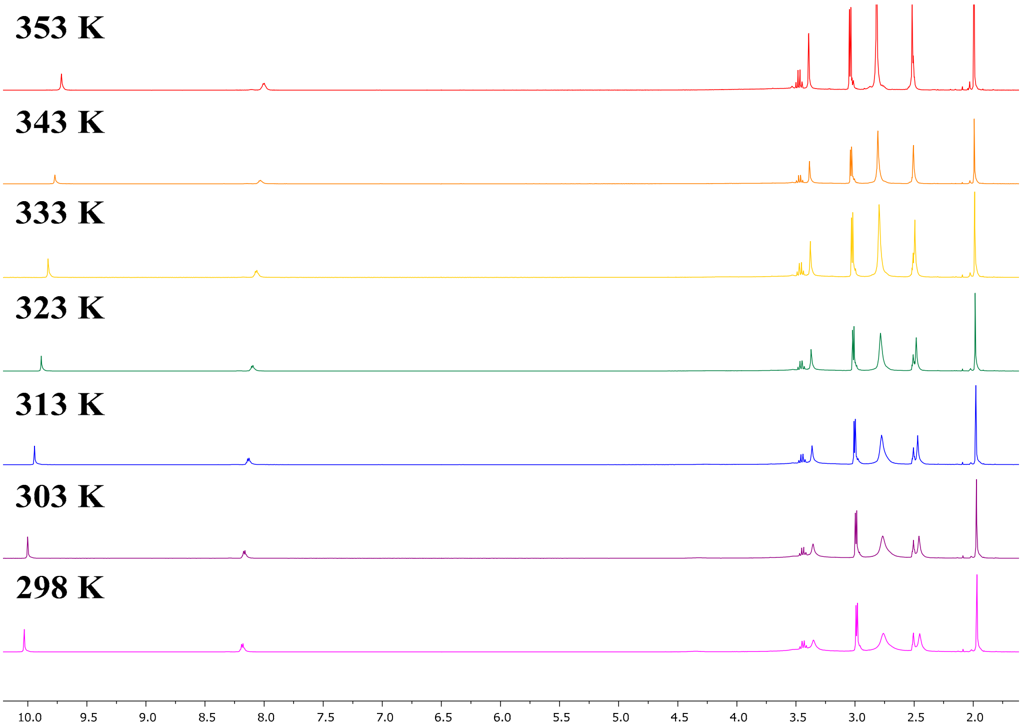

Figure S3. The variable-temperature ^{1}H NMR spectrum of H_2**L**. The lowest temperature (shown in pink) is at 25 °C, followed by 30, 40, 50, 60, 70 and 80 °C (shown in red).

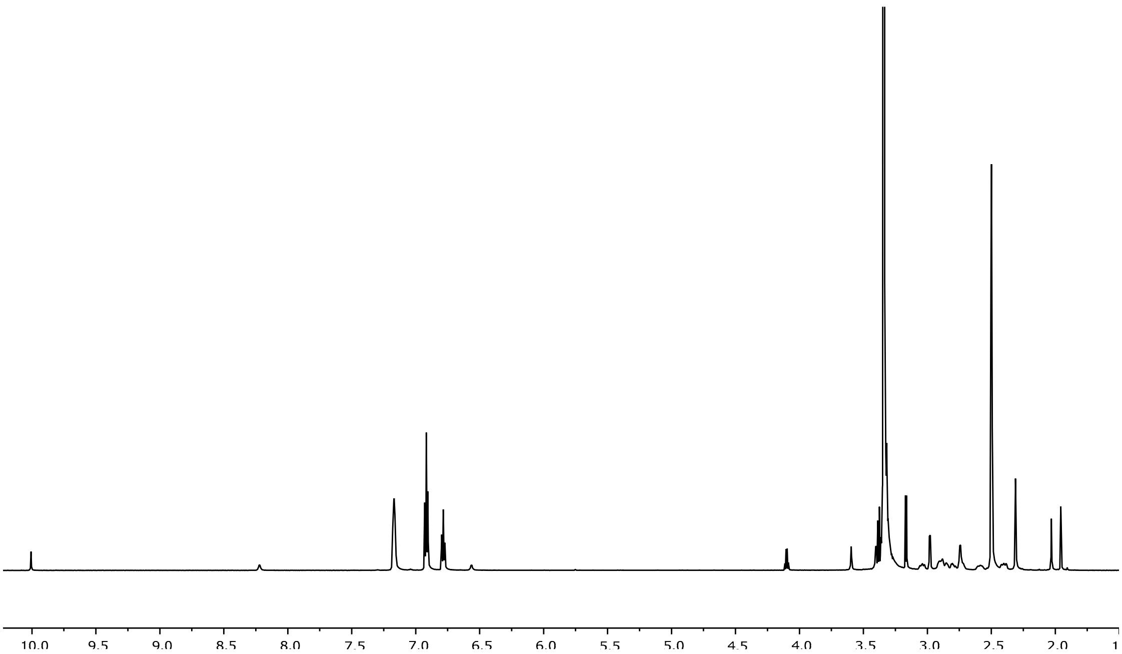

Figure S4. The ^{1}H NMR spectrum of [ZnH**L**][BPh_4] in d_6-DMSO.

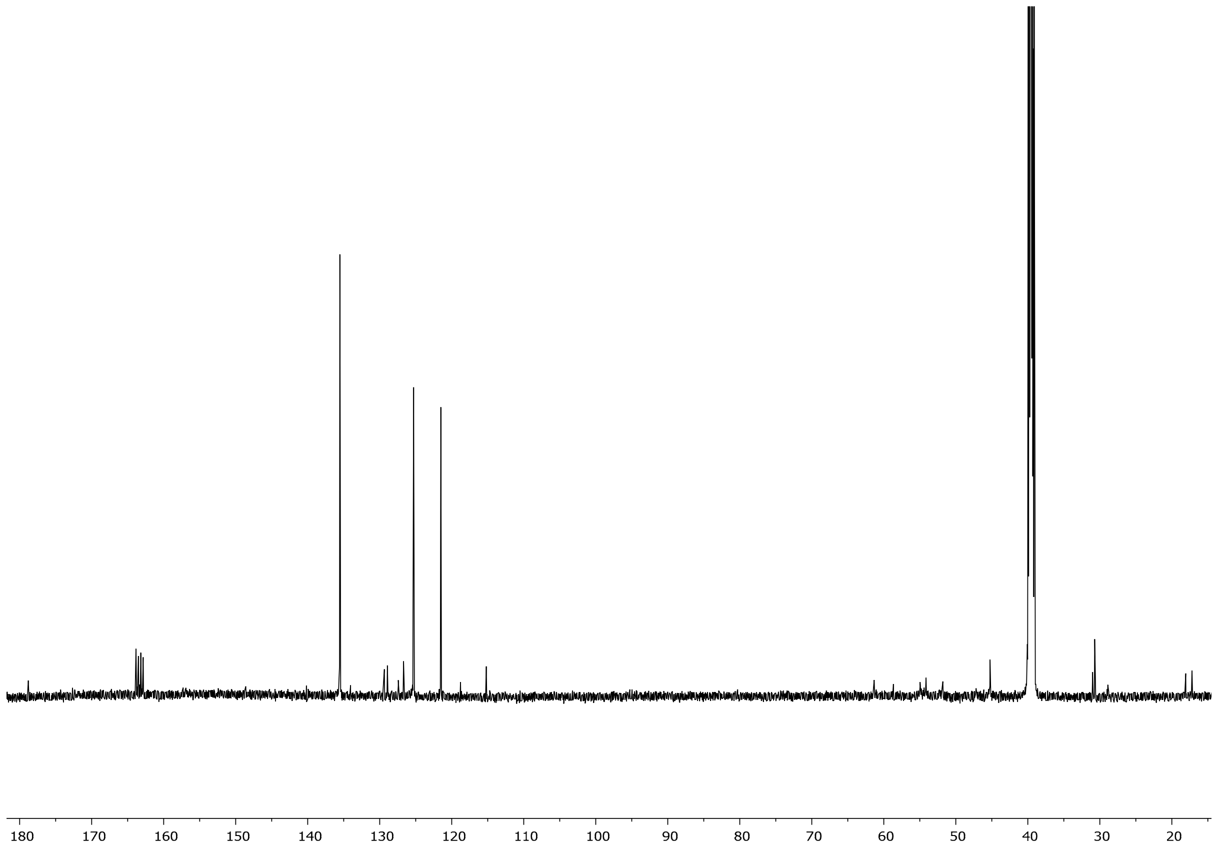

Figure S5. The $^{13}C\{^1H\}$ NMR spectrum of [ZnH**L**][BPh_4] in d_6-DMSO.

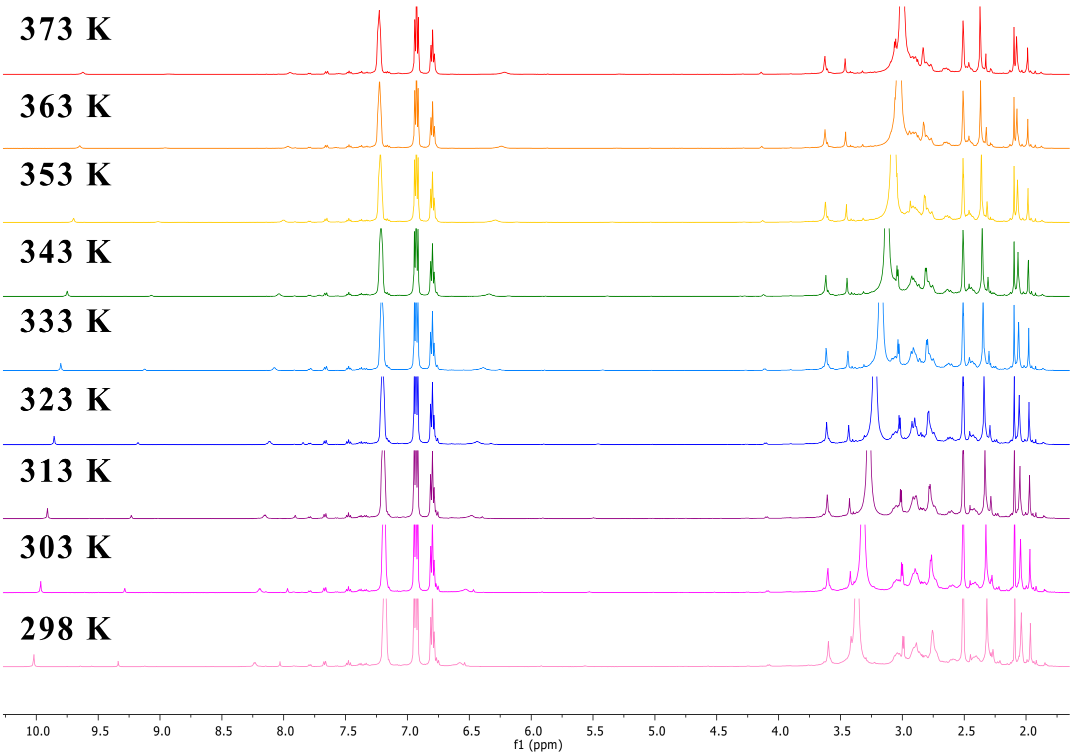

Figure S6. The variable-temperature ^{1}H NMR spectrum of [ZnH**L**][BPh_4]. The lowest temperature (shown in pink) is at 25 °C, followed by 30, 40, 50, 60, 70, 80, 90 and 100 °C (shown in red).

Mass Spectrometry Data

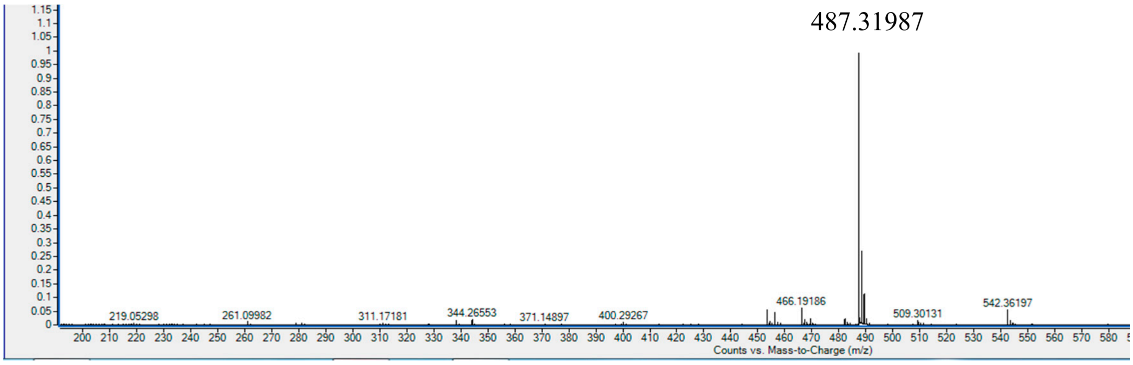

Figure S7. The high-resolution mass spectrum of H_2**L**. $[M + H]^+$: m/z = 487.31987.

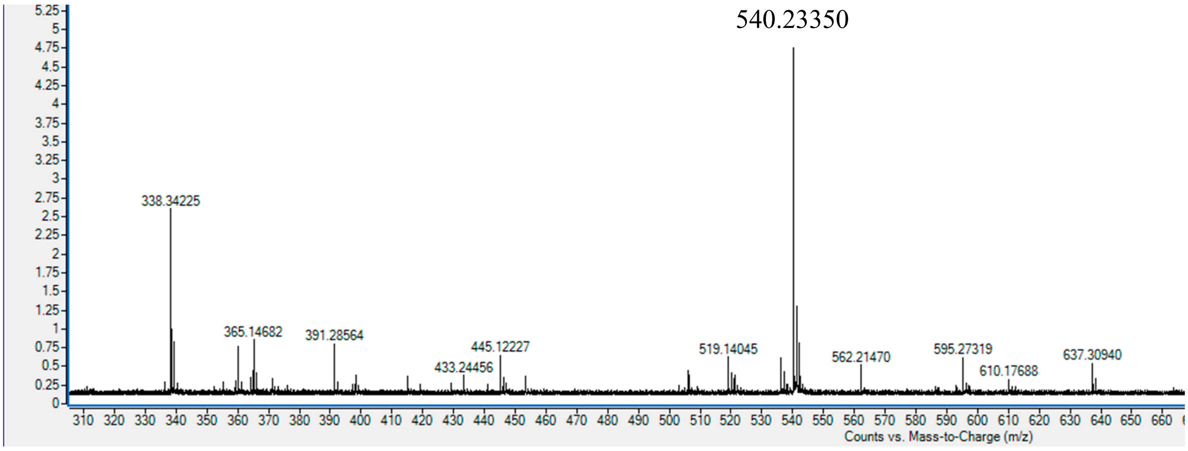

Figure S8. The high-resolution mass spectrum of [MnH**L**]$^{+}$. [M]$^{+}$: m/z = 540.23350.

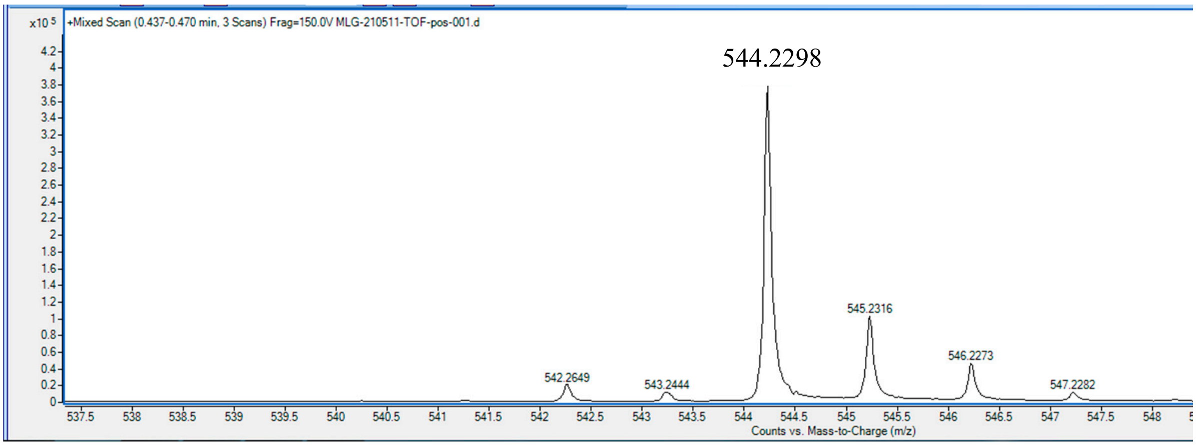

Figure S9. The high-resolution mass spectrum of [CoH**L**]$^{+}$. [M]$^{+}$: m/z = 544.2298.

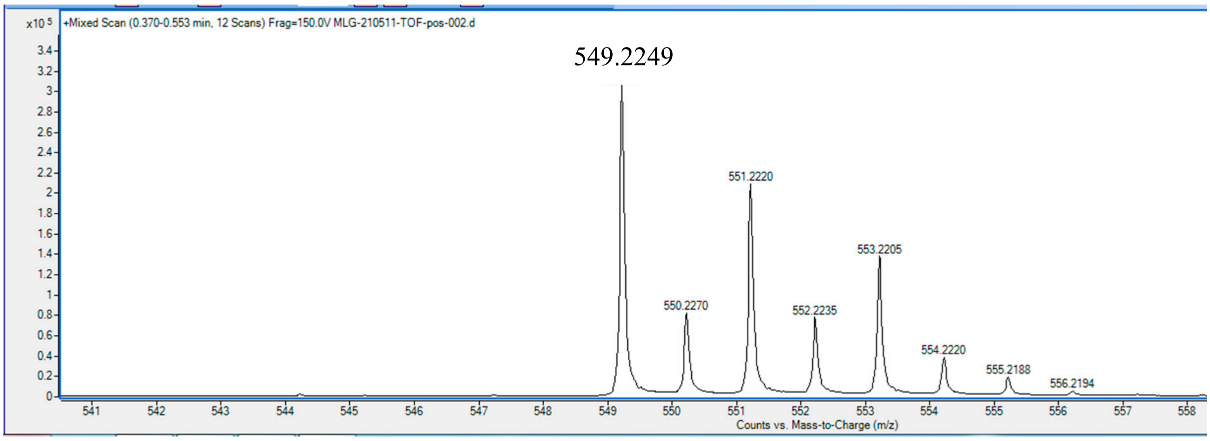

Figure S10. The high-resolution mass spectrum of [ZnH**L**]$^{+}$. [M]$^{+}$: m/z = 549.2249.

Analytical RP-HPLC Traces

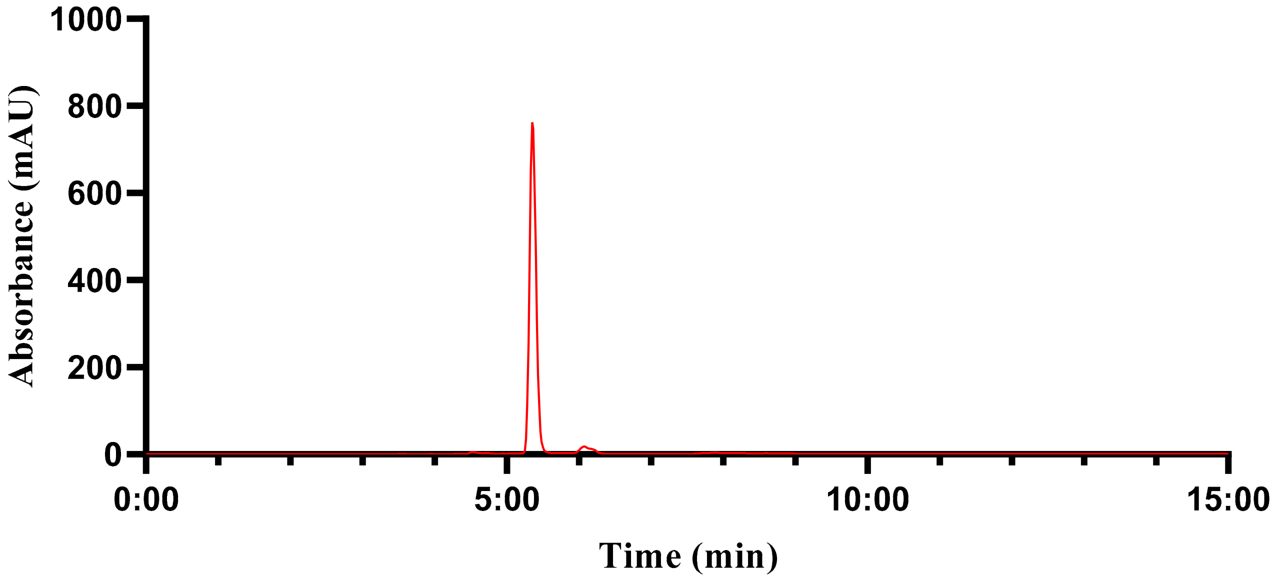

Figure S11. The analytical RP-HPLC trace of H_2**L**.

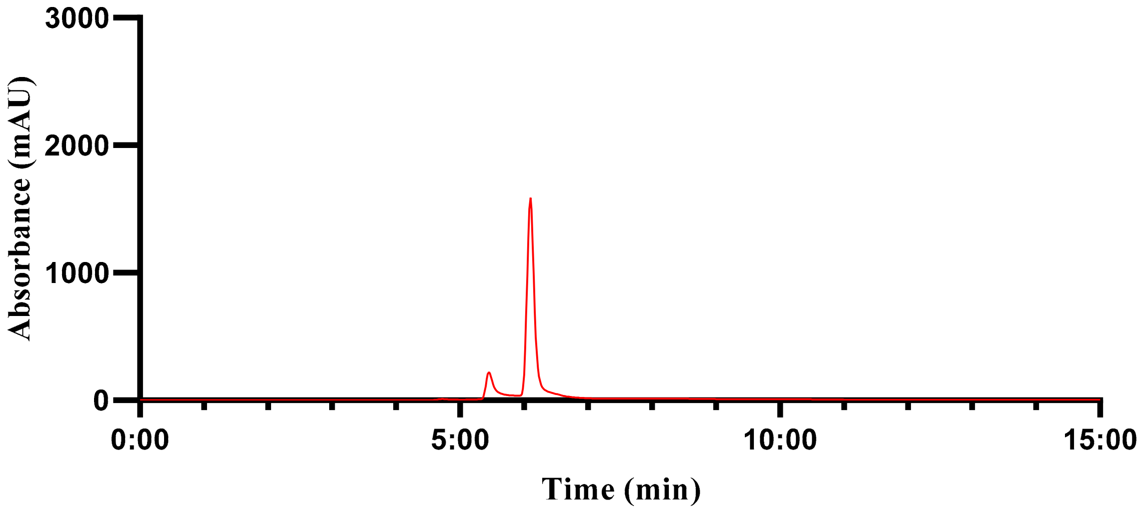

Figure S12. The analytical RP-HPLC trace of [MnH**L**]$^+$.

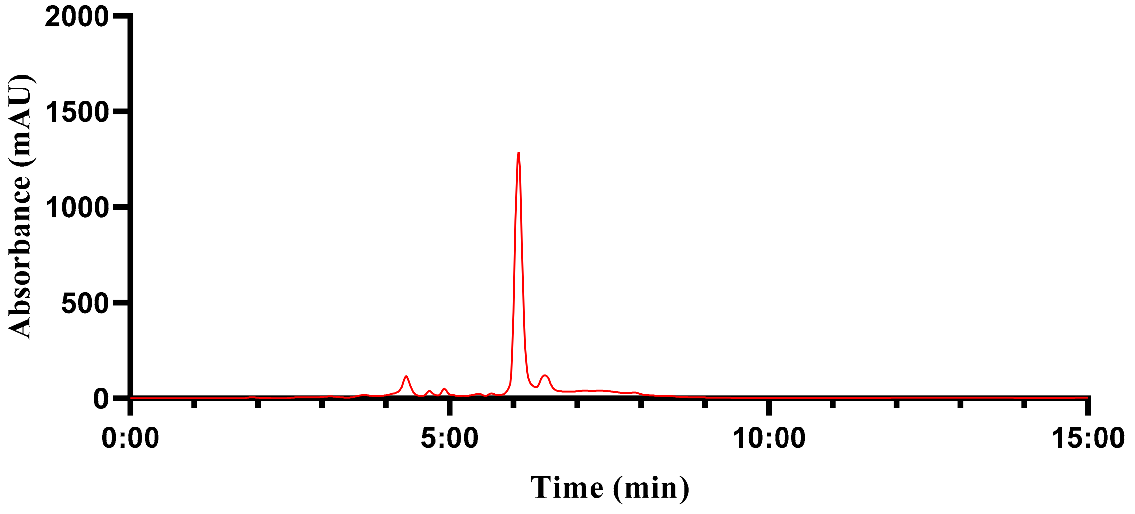

Figure S13. The analytical RP-HPLC trace of [CoH**L**]$^{+}$.

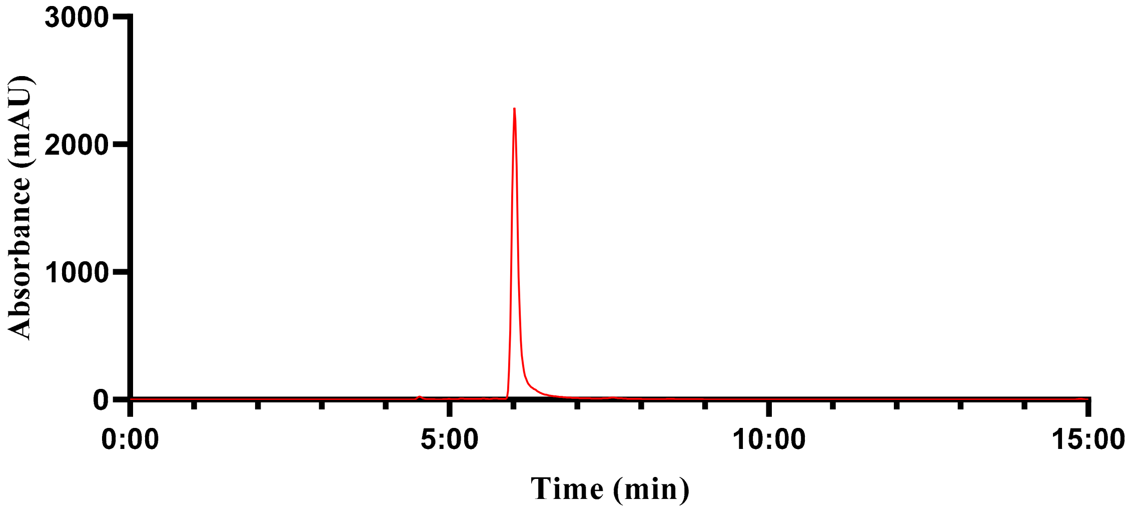

Figure S14. The analytical RP-HPLC trace of [ZnH**L**]$^{+}$.

Radiochemistry Data

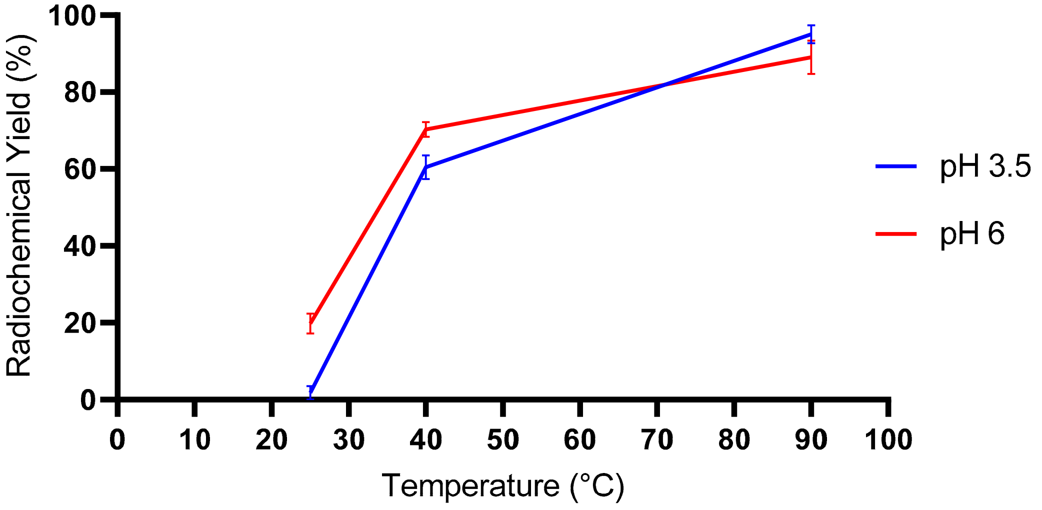

Figure S15. The radiochemical yield of [^{68}Ga][GaH**L**^{1}] at pH 3.5 and pH 6 at 25, 40 and 90 °C.

Table S1. The radiochemical yields for the reaction of ^{68}Ga with H_2**L** (0.5, 5, 50 and 500 µM) at pH 3.5 and pH 6 and different temperatures (25, 40 and 90 °C).

	**pH 3.5**	**pH 6**			
**Temperature (°C)**	**50 µM**	**0.5 µM**	**5 µM**	**50 µM**	**500 µM**
**25**	1.8±1.7	0.6±0.2	1.2±1.1	19.8±2.6	65.3±2.3
**40**	60.4±3.1	1.5±0.7	1.2±0.4	70.3±1.9	95.2±2.1
**90**	95.1±2.3	3.2±0.9	46.3±1.8	89.0±4.3	96.5±1.3

DFT Calculated Structures—Selected Bond Lengths, Bond Angles & Torsion Angles

Co^{2+} Δ(δ,δ,δ,δ) isomer

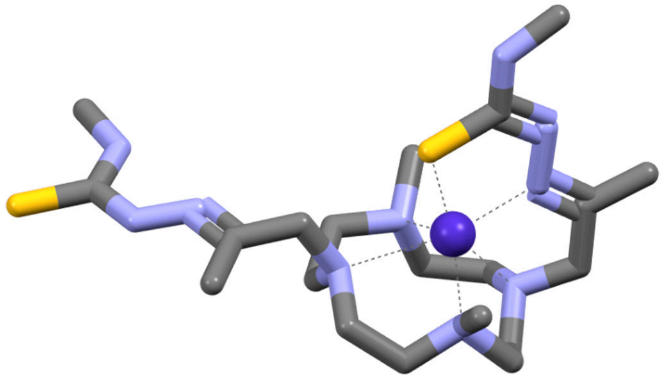

Figure S16. Optimised structure of Co^{2+} Δ(δ,δ,δ,δ) isomer at the B3LYP/TZVP level of theory.

Table S2. Selected calculated bond lengths (Å*)* for the Co^{2+} Δ(δ,δ,δ,δ) isomer at the B3LYP/TZVP level of theory without counterpoise correction for BSSE.

Bond lengths (Å)			
Co1-N4	2.217	C16-N8	1.285
Co1-N5	2.164	N5-N6	1.378
Co1-N2	2.248	N8-N9	1.364
Co1-S1	2.401	N6-C12	1.310
Co1-N1	2.351	N9-C18	1.369
Co1-N3	2.680	C12-S1	1.775
C10-N5	1.286	C18-S2	1.703

Table S3. Selected calculated bond angles (˚) for the Co^{2+} Δ(δ,δ,δ,δ) isomer at the B3LYP/TZVP level of theory without counterpoise correction for BSSE.

Bond angles (˚)			
S1-Co1-N1	153.95	N4-Co1-N1	78.61
S1-Co1-N3	83.04	N4-Co1-N3	74.17
S1-Co1-N4	114.12	N5-Co1-N3	163.16
N5-Co1-S1	80.13	N4-Co1-N5	113.26
N2-Co1-S1	108.26	N4-Co1-N2	123.46
C12-S1-Co1	96.43	N5-Co1-N2	109.28
N5-Co1-N1	73.88	N2-Co1-N3	75.76
N2-Co1-N1	79.31	N1-Co1-N3	122.93

Table S4. Calculated torsion angles (˚) for the Co^{2+} Δ(δ,δ,δ,δ) isomer at the B3LYP/TZVP level of theory without counterpoise correction for BSSE.

Torsion angles (Co^{2+}metal centre) (˚)		*Torsion angles (macrocycle) (˚)*	
S1-N5	40.67	N1-N4	54.41
N1-N2	13.42	N4-N3	64.53
N3-N4	22.41	N3-N2	56.29
Average	25.50	N2-N1	58.46

Co^{2+} distance from N2-N3-S1 plane = 1.254 Å.

Co^{2+} distance from N1-N4-N5 plane = 1.253 Å.

Co^{2+} distance from N11-N14-N13-N12 plane = 1.122 Å.

Co^{2+} Λ(λ,λ,λ,λ) isomer

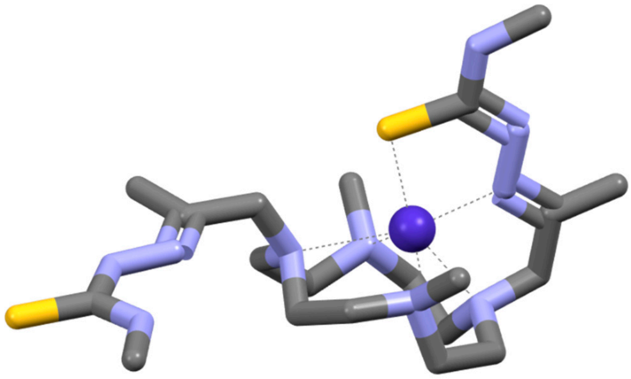

Figure S17. Optimised structure of Co^{2+} Λ(λ,λ,λ,λ) isomer at the B3LYP/TZVP level of theory.

Table S5. Selected calculated bond lengths (Å*)* for the Co^{2+} Λ(λ,λ,λ,λ) isomer at the B3LYP/TZVP level of theory without counterpoise correction for BSSE.

Bond lengths (Å)			
Co1-N4	2.229	C16-N8	1.286
Co1-N5	2.151	N5-N6	1.376
Co1-N2	2.229	N8-N9	1.352
Co1-S1	2.406	N6-C12	1.311
Co1-N1	2.365	N9-C18	1.379
Co1-N3	2.677	C12-S1	1.772
C10-N5	1.288	C18-S2	1.693

Table S6. Selected calculated bond angles (˚) for the Co^{2+} Λ(λ,λ,λ,λ) isomer at the B3LYP/TZVP level of theory without counterpoise correction for BSSE.

Bond angles (˚)			
S1-Co1-N1	154.03	N4-Co1-N1	78.73
S1-Co1-N3	83.42	N4-Co1-N3	75.72
S1-Co1-N4	107.69	N5-Co1-N3	163.22
N5-Co1-S1	80.21	N4-Co1-N5	105.83
N2-Co1-S1	115.45	N4-Co1-N2	122.61
C12-S1-Co1	96.02	N5-Co1-N2	116.94
N5-Co1-N1	73.85	N2-Co1-N3	73.68
N2-Co1-N1	78.48	N1-Co1-N3	122.36

Table S7. Calculated torsion angles (˚) for the Co^{2+} Λ(λ,λ,λ,λ) isomer at the B3LYP/TZVP level of theory without counterpoise correction for BSSE.

Torsion angles (Co^{2+}metal centre) (˚)		*Torsion angles (macrocycle) (˚)*	
S1-N5	-38.77	N1-N4	-54.32
N1-N2	-12.02	N4-N3	-64.51
N3-N4	-22.67	N3-N2	-56.73
Average	-24.49	N2-N1	-59.53

Distance from N2-N3-S1 plane = 1.230 Å.

Distance from N1-N4-N5 plane = 1.299 Å.

Distance from N11-N14-N13-N12 plane = 1.136 Å.

Co^{2+} Δ(λ,λ,λ,δ) isomer

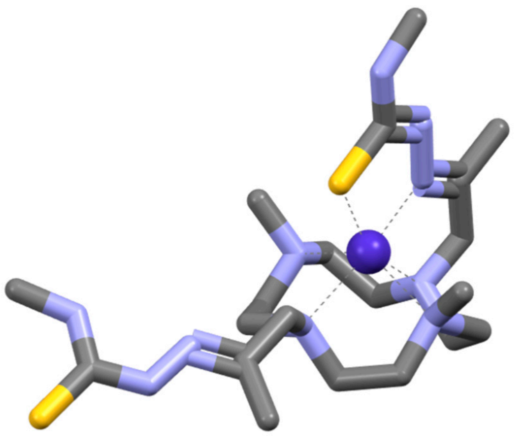

Figure S18. Optimised structure of Co^{2+} Δ(λ,λ,λ,δ) isomer at the B3LYP/TZVP level of theory.

Table S8. Selected calculated bond lengths (Å) for the Co^{2+} Δ(λ,λ,λ,δ) isomer at the B3LYP/TZVP level of theory without counterpoise correction for BSSE.

Bond lengths (Å)			
Co2-N14	2.239	C36-N18	1.284
Co2-N15	2.128	N15-N16	1.380
Co2-N12	2.259	N18-N19	1.362
Co2-S3	2.412	N16-C32	1.311
Co2-N11	2.402	N19-C38	1.370
Co2-N13	2.400	C32-S3	1.774
C30-N15	1.285	C38-S4	1.703

Table S9. Selected calculated bond angles (°) for the Co^{2+} Δ(λ,λ,λ,δ) isomer at the B3LYP/TZVP level of theory without counterpoise correction for BSSE.

Bond angles (°)			
S3-Co2-N11	156.50	N14-Co2-N11	77.55
S3-Co2-N13	90.21	N14-Co2-N13	78.54
S3-Co2-N14	108.80	N15-Co2-N13	171.15
N15-Co2-S3	80.94	N14-Co2-N15	104.12
N12-Co2-S3	108.51	N14-Co2-N12	135.59
C32-S3-Co2	95.33	N15-Co2-N12	104.74
N15-Co2-N11	75.56	N12-Co2-N13	78.09
N12-Co2-N11	77.86	N11-Co2-N13	113.29

Table S10. Calculated torsion angles (°) for the Co^{2+} Δ(λ,λ,λ,δ) isomer at the B3LYP/TZVP level of theory without counterpoise correction for BSSE.

Torsion angles (Co^{2+}metal centre) (°)		*Torsion angles (macrocycle) (°)*	
S3-N15	43.89	N11-N14	-57.04
N11-N12	22.17	N14-N13	-53.12
N13-N14	31.59	N13-N12	-50.92
Average	32.55	N12-N11	59.46

Distance from N12-N13-S3 plane = 1.265 Å.

Distance from N11-N14-N15 plane = 1.317 Å.

Distance from N11-N14-N13-N12 plane = 1.085 Å.

Co^{2+} Λ(δ,δ,δ,λ) isomer

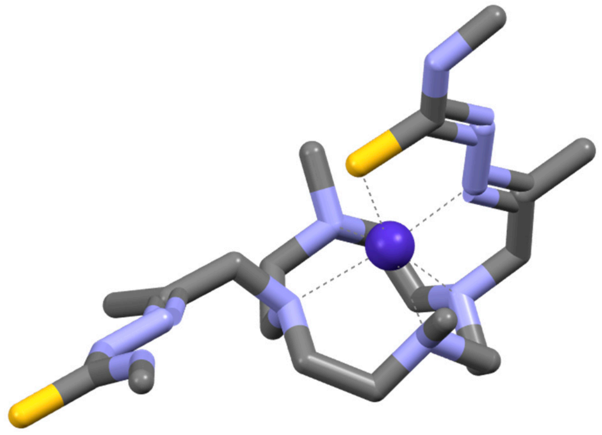

Figure S19. Optimised structure of Co^{2+} Λ(δ,δ,δ,λ) isomer at the B3LYP/TZVP level of theory.

Table S11. Selected calculated bond lengths (Å*)* for the Co^{2+} Λ(δ,δ,δ,λ) isomer at the B3LYP/TZVP level of theory without counterpoise correction for BSSE.

Bond lengths (Å)			
Co2-N14	2.239	C36-N18	1.284
Co2-N15	2.128	N15-N16	1.380
Co2-N12	2.259	N18-N19	1.362
Co2-S3	2.412	N16-C32	1.311
Co2-N11	2.402	N19-C38	1.370
Co2-N13	2.400	C32-S3	1.774
C30-N15	1.285	C38-S4	1.703

Table S12. Selected calculated bond angles (˚) for the Co^{2+} Λ(δ,δ,δ,λ) isomer at the B3LYP/TZVP level of theory without counterpoise correction for BSSE.

Bond angles (˚)			
S3-Co2-N11	156.50	N14-Co2-N11	77.55
S3-Co2-N13	90.21	N14-Co2-N13	78.54
S3-Co2-N14	108.80	N15-Co2-N13	171.15
N15-Co2-S3	80.94	N14-Co2-N15	104.12
N12-Co2-S3	108.51	N14-Co2-N12	135.59
C32-S3-Co2	95.33	N15-Co2-N12	104.74
N15-Co2-N11	75.56	N12-Co2-N13	78.09
N12-Co2-N11	77.86	N11-Co2-N13	113.29

Table S13. Calculated torsion angles (˚) for the Co^{2+} Λ(δ,δ,δ,λ) isomer at the B3LYP/TZVP level of theory without counterpoise correction for BSSE.

Torsion angles (Co^{2+}metal centre) (˚)		*Torsion angles (macrocycle) (˚)*	
S3-N15	-43.89	N11-N14	57.04
N11-N12	-22.17	N14-N13	53.12
N13-N14	-31.59	N13-N12	50.92
Average	-32.55	N12-N11	-59.46

Distance from N12-N13-S3 plane = 1.265 Å.

Distance from N11-N14-N15 plane = 1.317 Å.

Distance from N11-N14-N13-N12 plane = 1.085 Å.

Zn^{2+} Δ(δ,δ,δ,δ) isomer

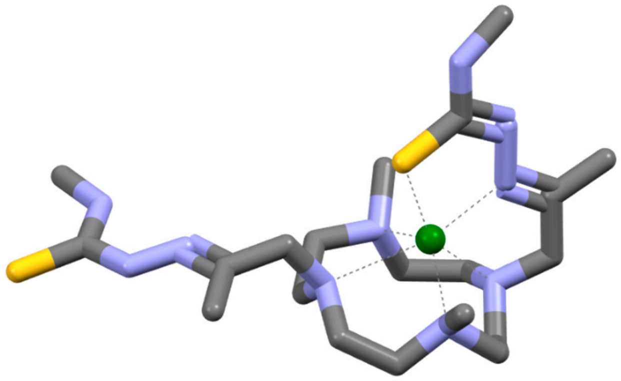

Figure S20. Optimised structure of Zn^{2+} Δ(δ,δ,δ,δ) isomer at the B3LYP/DGDZVP level of theory.

Table S14. Selected calculated bond lengths (Å*)* for the Zn^{2+} Δ(δ,δ,δ,δ) isomer at the B3LYP/TZVP level of theory without counterpoise correction for BSSE.

Bond lengths (Å)			
Zn1-N4	2.228	C16-N8	1.292
Zn1-N5	2.211	N5-N6	1.380
Zn1-N2	2.240	N8-N9	1.370
Zn1-S1	2.420	N6-C12	1.321
Zn1-N1	2.390	N9-C18	1.375
Zn1-N3	2.727	C12-S1	1.768
C10-N5	1.290	C18-S2	1.701

Table S15. Selected calculated bond angles (˚) for the Zn^{2+} Δ(δ,δ,δ,δ) isomer at the B3LYP/TZVP level of theory without counterpoise correction for BSSE.

Bond angles (˚)			
S1-Zn1-N1	152.39	N4-Zn1-N1	79.96
S1-Zn1-N3	83.73	N4-Zn1-N3	75.75
S1-Zn1-N4	107.62	N5-Zn1-N3	162.86
N5-Zn1-S1	79.52	N4-Zn1-N5	105.90
N2-Zn1-S1	114.69	N4-Zn1-N2	123.85
C12-S1-Zn1	96.69	N5-Zn1-N2	116.50
N5-Zn1-N1	72.69	N2-Zn1-N3	73.97
N2-Zn1-N1	79.37	N1-Zn1-N3	123.75

Table S16. Calculated torsion angles (˚) for the Zn^{2+} Δ(δ,δ,δ,δ) isomer at the B3LYP/TZVP level of theory without counterpoise correction for BSSE.

Torsion angles (Zn^{2+}metal centre) (˚)		*Torsion angles (macrocycle) (˚)*	
S1-N5	38.45	N1-N4	54.41
N1-N2	12.17	N4-N3	64.64
N3-N4	22.51	N3-N2	55.77
Average	24.38	N2-N1	58.80

Distance from N2-N3-S1 plane = 1.248 Å.

Distance from N1-N4-N5 plane = 1.315 Å.

Distance from N11-N14-N13-N12 plane = 1.121 Å.

Zn^{2+} Λ(λ,λ,λ,λ) isomer

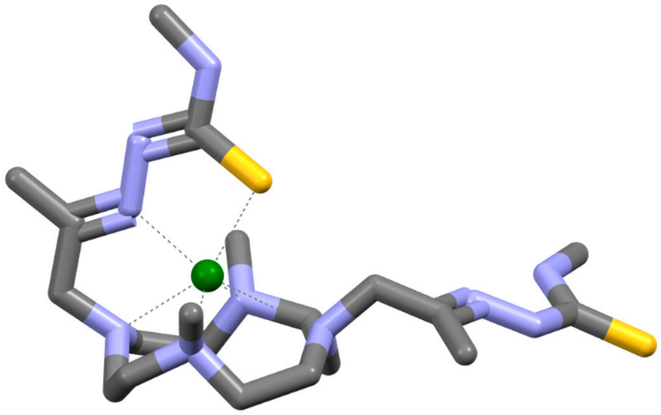

Figure S21. Optimised structure of Zn^{2+} Λ(λ,λ,λ,λ) isomer at the B3LYP/DGDZVP level of theory.

Table S17. Selected calculated bond lengths (Å) for the Zn^{2+} Λ(λ,λ,λ,λ) isomer at the B3LYP/TZVP level of theory without counterpoise correction for BSSE.

Bond lengths (Å)			
Zn1-N4	2.217	C16-N8	1.292
Zn1-N5	2.212	N5-N6	1.360
Zn1-N2	2.238	N8-N9	1.370
Zn1-S1	2.414	N6-C12	1.320
Zn1-N1	2.403	N9-C18	1.375
Zn1-N3	2.734	C12-S1	1.767
C10-N5	1.291	C18-S2	1.701

Table S18. Selected calculated bond angles (°) for the Zn^{2+} Λ(λ,λ,λ,λ) isomer at the B3LYP/TZVP level of theory without counterpoise correction for BSSE.

Bond angles (°)			
S1-Zn1-N1	152.45	N4-Zn1-N1	79.67
S1-Zn1-N3	83.78	N4-Zn1-N3	75.85
S1-Zn1-N4	108.63	N5-Zn1-N3	163.43
N5-Zn1-S1	79.69	N4-Zn1-N5	108.74
N2-Zn1-S1	113.81	N4-Zn1-N2	123.62
C12-S1-Zn1	96.81	N5-Zn1-N2	113.79
N5-Zn1-N1	72.80	N2-Zn1-N3	73.97
N2-Zn1-N1	79.42	N1-Zn1-N3	123.70

Table S19. Calculated torsion angles (°) for the Zn^{2+} Λ(λ,λ,λ,λ) isomer at the B3LYP/TZVP level of theory without counterpoise correction for BSSE.

Torsion angles (Zn^{2+} metal centre) (°)		*Torsion angles (macrocycle) (°)*	
S1-N5	-39.57	N1-N4	-59.42
N1-N2	-13.25	N4-N3	-55.45
N3-N4	-22.81	N3-N2	-64.71
Average	-25.21	N2-N1	-54.22

Distance from N2-N3-S1 plane = 1.259 Å.

Distance from N1-N4-N5 plane = 1.277 Å.

Distance from N11-N14-N13-N12 plane = 1.124 Å.

Zn^{2+} Δ(λ,λ,λ,λ) isomer

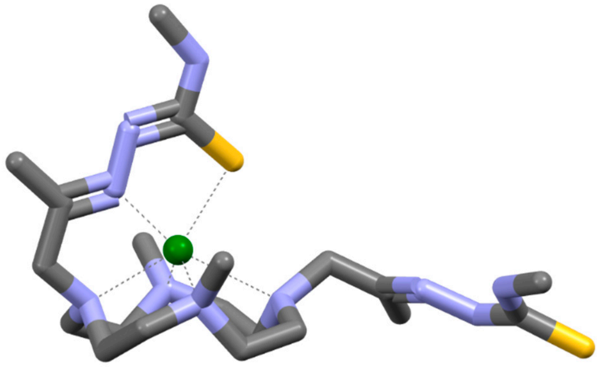

Figure S22. Optimised structure of Zn^{2+} Δ(λ,λ,λ,λ) isomer at the B3LYP/DGDZVP level of theory.

Table S20. Selected calculated bond lengths (Å*)* for the Zn^{2+} Δ(λ,λ,λ,λ) isomer at the B3LYP/TZVP level of theory without counterpoise correction for BSSE.

Bond lengths (Å)			
Zn2-N14	2.238	C36-N18	1.292
Zn2-N15	2.212	N15-N16	1.381
Zn2-N12	2.260	N18-N19	1.370
Zn2-S3	2.419	N16-C32	1.320
Zn2-N11	2.411	N19-C38	1.375
Zn2-N13	2.701	C32-S3	1.768
C30-N15	1.269	C38-S4	1.701

Table S21. Selected calculated bond angles (˚) for the Zn^{2+} Δ(λ,λ,λ,λ) isomer at the B3LYP/TZVP level of theory without counterpoise correction for BSSE.

Bond angles (˚)			
S3-Zn2-N11	151.87	N14-Zn2-N11	79.59
S3-Zn2-N13	84.47	N14-Zn2-N13	74.60
S3-Zn2-N14	113.66	N15-Zn2-N13	159.84
N15-Zn2-S3	79.72	N14-Zn2-N15	100.50
N12-Zn2-S3	109.68	N14-Zn2-N12	123.61
C32-S3-Zn2	96.58	N15-Zn2-N12	121.67
N15-Zn2-N11	73.30	N12-Zn2-N13	75.51
N12-Zn2-N11	78.86	N11-Zn2-N13	123.68

Table S22. Calculated torsion angles (˚) for the Zn^{2+} Δ(λ,λ,λ,λ) isomer at the B3LYP/TZVP level of theory without counterpoise correction for BSSE.

Torsion angles (Zn^{2+}metal centre) (˚)		*Torsion angles (macrocycle) (˚)*	
S3-N15	30.17	N11-N14	-61.26
N11-N12	8.87	N14-N13	-55.03
N13-N14	18.18	N13-N12	-64.59
Average	19.07	N12-N11	-55.21

Distance from N12-N13-S3 plane = 1.318 Å.

Distance from N11-N14-N15 plane = 1.383 Å.

Distance from N11-N14-N13-N12 plane = 1.130 Å.

Zn^{2+} Λ(δ,δ,δ,δ) isomer

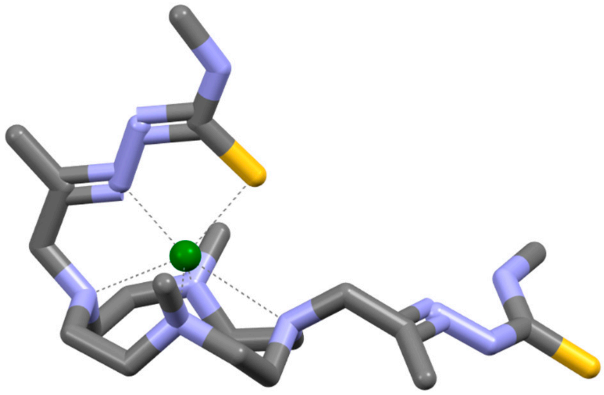

Figure S23. Optimised structure of Zn^{2+} Λ(δ,δ,δ,δ) isomer at the B3LYP/DGDZVP level of theory.

Table S23. Selected calculated bond lengths (Å*)* for the Zn^{2+} Λ(δ,δ,δ,δ) isomer at the B3LYP/TZVP level of theory without counterpoise correction for BSSE.

Bond lengths (Å)			
Zn2-N14	2.242	C36-N18	1.292
Zn2-N15	2.210	N15-N16	1.360
Zn2-N12	2.222	N18-N19	1.370
Zn2-S3	2.421	N16-C32	1.321
Zn2-N11	2.399	N19-C38	1.375
Zn2-N13	2.735	C32-S3	1.767
C30-N15	1.291	C38-S4	1.701

Table S24. Selected calculated bond angles (˚) for the Zn^{2+} Λ(δ,δ,δ,δ) isomer at the B3LYP/TZVP level of theory without counterpoise correction for BSSE.

Bond angles (˚)			
S3-Zn2-N11	152.38	N14-Zn2-N11	79.31
S3-Zn2-N13	83.94	N14-Zn2-N13	74.01
S3-Zn2-N14	114.24	N15-Zn2-N13	163.38
N15-Zn2-S3	79.56	N14-Zn2-N15	114.69
N12-Zn2-S3	108.23	N14-Zn2-N12	123.88
C32-S3-Zn2	96.64	N15-Zn2-N12	107.29
N15-Zn2-N11	72.84	N12-Zn2-N13	75.88
N12-Zn2-N11	79.62	N11-Zn2-N13	123.59

Table S25. Calculated torsion angles (˚) for the Zn^{2+} Λ(δ,δ,δ,δ) isomer at the B3LYP/TZVP level of theory without counterpoise correction for BSSE.

Torsion angles (Zn^{2+}metal centre) (˚)		*Torsion angles (macrocycle) (˚)*	
S3-N15	-39.72	N11-N14	59.25
N11-N12	-14.76	N14-N13	55.32
N13-N14	-20.59	N13-N12	65.04
Average	-25.02	N12-N11	54.28

Distance from N12-N13-S3 plane = 1.329 Å.

Distance from N11-N14-N15 plane = 1.196 Å.

Distance from N11-N14-N13-N12 plane = 1.123 Å.

DFT Calculated Structures—Cartesian Coordinates and Absolute Energies

Table S26. Cartesian coordinates for the Co^{2+} Δ(δ,δ,δ,δ) isomer.

E(RB3LYP) = -3513.941851 Hartree.

Atom	X	Y	Z
Co	-1.5892520	-0.2188170	-0.0420990
S	-0.9883350	1.8060110	-1.1846080
N	-3.0232500	-1.5808470	1.2298220
C	-2.9242550	-2.9322670	0.6443140
H	-2.0048830	-3.3966070	0.9970080
H	-3.7469750	-3.5749200	0.9791590
S	7.8836910	0.1110880	0.1691560
N	-1.8218160	-2.0012390	-1.3921570
C	-2.9364920	-2.8409040	-0.8729930
H	-3.8699110	-2.3900670	-1.2082750
H	-2.8954680	-3.8447250	-1.3092560
N	0.8976050	-0.9531540	-0.7180970
C	-0.5843610	-2.8272470	-1.4274540
H	-0.6810470	-3.5946700	-2.2049500
H	-0.5034600	-3.3550870	-0.4794700
N	-0.4960830	-0.2796690	1.8851070
C	0.6869690	-2.0376890	-1.6892900
H	1.5194530	-2.7531440	-1.7064000
H	0.6407270	-1.5895000	-2.6808570
N	-3.4201310	0.9069210	0.2100820
C	1.3405870	-1.4260050	0.6040660
H	2.4233890	-1.5910280	0.6363570
H	0.8819940	-2.3943860	0.7950870
N	-3.5396020	2.2152660	-0.2063840
C	0.9734060	-0.4403190	1.7022950
H	1.4300680	-0.7575640	2.6464810
H	1.3851270	0.5398470	1.4691020
N	-2.5388710	3.9646390	-1.2676280
C	-1.0206930	-1.4134840	2.6902560
H	-0.7107490	-1.3073690	3.7363030
H	-0.5690240	-2.3325660	2.3217210
N	4.0199670	0.2479090	-0.5396160
C	-2.5329680	-1.5120590	2.6245010
H	-2.8679990	-2.3800680	3.2040880
H	-2.9798290	-0.6340100	3.0879010
N	5.3565360	-0.0086540	-0.6322650
C	-4.4024780	-1.0613250	1.1466790
H	-4.9831030	-1.6522970	0.4329680
H	-4.9201300	-1.1825010	2.1048070
N	5.6918470	1.2421210	1.2576670
C	-4.4767110	0.3731340	0.7120050

C	-5.7908220	1.0744810	0.8412840
H	-6.1033810	1.4822230	-0.1215050
H	-6.5597430	0.3989360	1.2144610
H	-5.7029310	1.9207510	1.5271970
C	-2.4900430	2.6847390	-0.8346500
C	-3.6844130	4.8399000	-1.0907300
H	-3.9080100	4.9923870	-0.0326210
H	-3.4505740	5.8008480	-1.5443170
H	-4.5757240	4.4281530	-1.5695620
C	-2.1624500	-1.5427440	-2.7618310
H	-3.0729880	-0.9473250	-2.7260970
H	-1.3672150	-0.9174590	-3.1584560
H	-2.3177140	-2.3959290	-3.4312540
C	1.7635110	0.1285860	-1.2519380
H	1.3361180	0.4441150	-2.2040000
H	1.7052390	0.9775720	-0.5757430
C	3.2316830	-0.2046890	-1.4472410
C	3.6933830	-0.9871990	-2.6456130
H	4.0407810	-1.9865300	-2.3634340
H	2.8945850	-1.1050220	-3.3731040
H	4.5231050	-0.4798080	-3.1465740
C	6.2272700	0.4861520	0.3015220
C	6.4341520	1.8630820	2.3422430
H	7.1748000	2.5659540	1.9586190
H	5.7237200	2.3991370	2.9673670
H	6.9477110	1.1133120	2.9451190
C	-0.7470100	1.0045730	2.5860250
H	-0.2318670	1.0266470	3.5525170
H	-0.3883460	1.8240980	1.9668500
H	-1.8118340	1.1461290	2.7526780
H	-1.7322590	4.3138770	-1.7558010
H	5.7494970	-0.5986010	-1.3546170
H	4.6899680	1.3763510	1.2079910

Table S27. Cartesian coordinates for the Co^{2+} Λ(λ,λ,λ,λ) isomer.

E(RB3LYP) = -3513.940637 Hartree.

Atom	X	Y	Z
Co	1.6079370	-0.2143580	-0.1232930
S	1.2160990	2.0410980	-0.8638530
N	2.9152390	-1.9468360	0.8157980
C	2.6048050	-3.1542250	0.0312740
H	1.6515510	-3.5509530	0.3747220
H	3.3513090	-3.9426430	0.1851890
S	-7.9264360	0.1242380	-0.0410860
N	1.4770040	-1.7789420	-1.7061640

C	2.5261390	-2.7995000	-1.4441580
H	3.4773800	-2.3857370	-1.7767870
H	2.3444920	-3.7003510	-2.0407620
N	-0.9951690	-0.5180610	-0.6671360
C	0.1535840	-2.4532680	-1.7170700
H	0.0948040	-3.1227710	-2.5835420
H	0.0789960	-3.0822780	-0.8317180
N	0.6515620	-0.4463440	1.8768650
C	-1.0139980	-1.4852130	-1.7757880
H	-1.9419070	-2.0718070	-1.8040840
H	-0.9715300	-0.9258750	-2.7081000
N	3.5487200	0.6354290	0.2492000
C	-1.4302250	-1.1018220	0.6163720
H	-2.5194350	-1.0887020	0.7131160
H	-1.1304810	-2.1483890	0.6412380
N	3.7998700	1.9803890	0.1062160
C	-0.8323430	-0.3503360	1.7963630
H	-1.2746630	-0.7180880	2.7297730
H	-1.0822090	0.7067920	1.7207300
N	2.9755500	4.0063010	-0.5233070
C	1.0417120	-1.7625320	2.4385400
H	0.8033730	-1.8045410	3.5077630
H	0.4467940	-2.5331090	1.9526660
N	-4.0465400	0.5401230	-0.4068000
C	2.5185410	-2.0576530	2.2363380
H	2.7450960	-3.0557520	2.6299350
H	3.1208230	-1.3550730	2.8094130
N	-5.3589600	0.3415160	-0.6607730
C	4.3355230	-1.5765170	0.7052370
H	4.7785680	-2.0487390	-0.1770090
H	4.9086430	-1.9644620	1.5551550
N	-5.8232060	0.5722080	1.5790820
C	4.5542940	-0.0955020	0.5862420
C	5.9350030	0.4298970	0.8156410
H	6.2508080	1.0650870	-0.0124660
H	6.6470480	-0.3850300	0.9430920
H	5.9567180	1.0531640	1.7143740
C	2.7990510	2.6730860	-0.3801080
C	4.1975990	4.7079480	-0.1700130
H	4.4530420	4.5544970	0.8803480
H	4.0409410	5.7701550	-0.3453100
H	5.0405090	4.3681080	-0.7765840
C	1.7354710	-1.1495770	-3.0279390
H	2.7333420	-0.7160910	-3.0266990
H	1.0209920	-0.3530900	-3.2151260
H	1.6689200	-1.8889700	-3.8337310
C	-1.7504660	0.7253850	-1.0171070

H	-1.2259140	1.1904700	-1.8501050
H	-1.6807230	1.4033650	-0.1699800
C	-3.2097450	0.5440420	-1.3835850
C	-3.6207550	0.3713800	-2.8223730
H	-4.0231310	-0.6287580	-3.0145170
H	-2.7813580	0.5246540	-3.4964520
H	-4.3976240	1.0930070	-3.0931650
C	-6.2962320	0.3580720	0.3509910
C	-6.6437520	0.6781550	2.7733080
H	-7.3831330	1.4735840	2.6725870
H	-5.9875540	0.9036930	3.6107770
H	-7.1667250	-0.2580020	2.9738210
C	1.1383550	0.6472590	2.7567900
H	0.6790220	0.5778870	3.7491080
H	0.8787130	1.6026130	2.3061940
H	2.2180000	0.6010740	2.8637120
H	2.1999430	4.5337910	-0.8853480
H	-5.7133140	0.1472630	-1.5895970
H	-4.8229100	0.7073660	1.6507110

Table S28. Cartesian coordinates for the Co^{2+} Δ(λ,λ,λ,δ) isomer.

E(RB3LYP) = -3513.939029 Hartree.

Atom	X	Y	Z
Co	-1.4491940	-0.1576430	0.0262090
S	-0.7922950	2.0011040	-0.8273820
S	7.8435450	0.0241000	0.0240740
N	-2.9560230	-1.8273940	0.8694600
N	-1.5692500	-1.6062380	-1.7030620
N	0.8198490	-0.9225060	-0.1375780
N	-0.8667750	-0.2877470	2.1845310
N	-3.3463630	0.8066270	0.0577030
N	-3.5075910	2.1248220	-0.3188370
N	-2.5055190	4.0177780	-1.0900250
N	3.9541180	0.0834170	-0.5267670
N	5.2768110	-0.2332180	-0.5979300
N	5.7318640	1.4784760	0.8562560
C	-2.8459520	-2.9762440	-0.0532150
H	-1.9603810	-3.5509450	0.2124670
H	-3.7016040	-3.6548250	0.0417340
C	-2.7476950	-2.4874800	-1.4854400
H	-3.6378000	-1.9100610	-1.7366350
H	-2.7182230	-3.3418220	-2.1702470
C	-0.3005000	-2.3944380	-1.8055940
H	0.2492670	-2.0300780	-2.6715860
H	-0.5234670	-3.4447650	-2.0094150

C	0.5822540	-2.3168140	-0.5630320
H	0.1036780	-2.8380120	0.2631600
H	1.5155750	-2.8567210	-0.7582010
C	1.3833670	-0.8273460	1.2287460
H	1.7746100	0.1817380	1.3458900
H	2.2311170	-1.5083730	1.3641880
C	0.3588600	-1.1164290	2.3128550
H	0.0737240	-2.1648720	2.2820000
H	0.8257590	-0.9511700	3.2907280
C	-1.9888870	-0.9023830	2.9392190
H	-2.7772860	-0.1533490	3.0198120
H	-1.6762290	-1.1526800	3.9596740
C	-2.5236470	-2.1441230	2.2462020
H	-1.7577320	-2.9154680	2.1973880
H	-3.3433460	-2.5634930	2.8396720
C	-4.3167260	-1.2449980	0.8522060
H	-4.9656350	-1.8343700	0.1954930
H	-4.7719730	-1.3324840	1.8447110
C	-4.4127680	0.1905120	0.4251710
C	-5.7719820	0.8151890	0.4519790
H	-6.0366500	1.1937350	-0.5371330
H	-6.5265980	0.0983720	0.7740570
H	-5.7833430	1.6716520	1.1296050
C	-2.4103250	2.7212990	-0.7171810
C	-3.7424660	4.7787840	-1.0794270
H	-4.1646400	4.8358560	-0.0736660
H	-3.5245280	5.7858710	-1.4288560
H	-4.4909440	4.3294950	-1.7360570
C	-1.7887730	-0.8439460	-2.9585830
H	-2.6905430	-0.2417590	-2.8640930
H	-0.9518760	-0.1738630	-3.1360660
H	-1.9001080	-1.5187650	-3.8142980
C	1.6644850	-0.1460610	-1.0912080
H	1.1873000	-0.1653450	-2.0690170
H	1.6591160	0.8851360	-0.7471570
C	3.1132870	-0.5788170	-1.2360500
C	3.5057830	-1.6722930	-2.1923380
H	3.8956130	-2.5495720	-1.6663700
H	2.6641040	-1.9930110	-2.8006090
H	4.2873200	-1.3258480	-2.8756250
C	6.2037150	0.4734270	0.1224620
C	6.5417900	2.3516320	1.6894140
H	7.2783320	2.8902940	1.0920030
H	5.8763500	3.0672630	2.1665250
H	7.0673630	1.7831060	2.4575090
C	-0.6204810	1.0735040	2.7147650
H	-0.3849080	1.0349870	3.7842130

H	0.2039690	1.5408430	2.1834570
H	-1.5079210	1.6862640	2.5672980
H	-1.6619360	4.4668080	-1.4025670
H	5.6233190	-1.0095160	-1.1472580
H	4.7315210	1.6269080	0.8165650

Table S29. Cartesian coordinates for the Co^{2+} $\Lambda(\delta,\delta,\delta,\lambda)$ isomer.

E(RB3LYP) = -3513.939029 Hartree.

Atom	X	Y	Z
Co	1.4491940	-0.1576430	0.0262100
S	0.7922950	2.0011040	-0.8273810
S	-7.8435450	0.0241000	0.0240740
N	2.9560230	-1.8273950	0.8694600
N	1.5692500	-1.6062380	-1.7030620
N	-0.8198490	-0.9225060	-0.1375780
N	0.8667750	-0.2877480	2.1845310
N	3.3463640	0.8066270	0.0577030
N	3.5075910	2.1248220	-0.3188370
N	2.5055190	4.0177780	-1.0900250
N	-3.9541180	0.0834170	-0.5267670
N	-5.2768110	-0.2332180	-0.5979310
N	-5.7318640	1.4784750	0.8562570
C	2.8459520	-2.9762440	-0.0532160
H	1.9603800	-3.5509450	0.2124660
H	3.7016040	-3.6548260	0.0417330
C	2.7476950	-2.4874800	-1.4854400
H	3.6378000	-1.9100610	-1.7366360
H	2.7182230	-3.3418220	-2.1702480
C	0.3005000	-2.3944380	-1.8055940
H	-0.2492670	-2.0300770	-2.6715860
H	0.5234660	-3.4447640	-2.0094160
C	-0.5822540	-2.3168140	-0.5630320
H	-0.1036780	-2.8380120	0.2631600
H	-1.5155760	-2.8567210	-0.7582010
C	-1.3833670	-0.8273460	1.2287460
H	-1.7746100	0.1817390	1.3458900
H	-2.2311180	-1.5083720	1.3641880
C	-0.3588600	-1.1164300	2.3128550
H	-0.0737240	-2.1648730	2.2820000
H	-0.8257590	-0.9511700	3.2907280
C	1.9888870	-0.9023830	2.9392190
H	2.7772860	-0.1533500	3.0198120
H	1.6762290	-1.1526810	3.9596740
C	2.5236470	-2.1441240	2.2462020
H	1.7577320	-2.9154680	2.1973880

H	3.3433450	-2.5634940	2.8396720
C	4.3167260	-1.2449980	0.8522060
H	4.9656350	-1.8343710	0.1954930
H	4.7719730	-1.3324840	1.8447110
C	4.4127680	0.1905110	0.4251710
C	5.7719820	0.8151890	0.4519790
H	6.0366500	1.1937340	-0.5371330
H	6.5265980	0.0983710	0.7740560
H	5.7833440	1.6716510	1.1296050
C	2.4103250	2.7212990	-0.7171810
C	3.7424670	4.7787840	-1.0794270
H	4.1646400	4.8358560	-0.0736660
H	3.5245290	5.7858710	-1.4288560
H	4.4909450	4.3294940	-1.7360570
C	1.7887730	-0.8439450	-2.9585830
H	2.6905430	-0.2417580	-2.8640930
H	0.9518770	-0.1738620	-3.1360660
H	1.9001080	-1.5187640	-3.8142980
C	-1.6644850	-0.1460600	-1.0912070
H	-1.1873000	-0.1653440	-2.0690170
H	-1.6591160	0.8851370	-0.7471560
C	-3.1132870	-0.5788170	-1.2360500
C	-3.5057830	-1.6722920	-2.1923390
H	-3.8956130	-2.5495710	-1.6663710
H	-2.6641040	-1.9930100	-2.8006100
H	-4.2873200	-1.3258460	-2.8756260
C	-6.2037150	0.4734270	0.1224620
C	-6.5417900	2.3516310	1.6894150
H	-7.2783330	2.8902930	1.0920040
H	-5.8763500	3.0672620	2.1665260
H	-7.0673630	1.7831040	2.4575100
C	0.6204810	1.0735030	2.7147650
H	0.3849080	1.0349870	3.7842140
H	-0.2039690	1.5408430	2.1834580
H	1.5079210	1.6862640	2.5672980
H	1.6619360	4.4668080	-1.4025670
H	-5.6233190	-1.0095150	-1.1472590
H	-4.7315220	1.6269070	0.8165660

Table S30. Cartesian coordinates for the Zn^{2+} Λ(δ,δ,δ,δ) isomer.

E(RB3LYP) = -3909.803671 Hartree.

Atom	X	Y	Z
Zn	1.5575290	-0.1926820	0.0633370
S	-8.0030160	0.0644040	0.1386470

S	0.8267530	2.0353570	-0.5406040
N	3.2351490	-1.7701140	0.7368820
N	3.4426150	0.9314280	0.3233020
N	-5.4619890	0.0006800	-0.6388810
H	-5.8318680	-0.6210450	-1.3528990
N	-5.8482400	1.2509510	1.2550000
H	-4.8438930	1.4021160	1.2199480
N	0.6554420	-0.9743710	1.9616110
N	3.5450580	2.2612530	-0.0306360
N	1.7532840	-1.3886960	-1.7993800
N	-1.0042890	-0.8873470	-0.5533260
N	-4.1245890	0.2696410	-0.5126660
C	-6.3596070	0.4762100	0.2884470
N	2.4661220	4.1038430	-0.8501580
H	1.5841470	4.5495800	-1.0610010
C	-3.3091650	-0.1625740	-1.4166880
C	4.4893720	0.3921050	0.8515240
C	-1.8413320	0.1731120	-1.1709660
H	-1.3784740	0.4715250	-2.1144320
H	-1.8093890	1.0397060	-0.5080750
C	2.4148930	2.8044190	-0.4447150
C	-6.6204710	1.8604220	2.3317150
H	-7.3854700	2.5328600	1.9332040
H	-5.9315490	2.4322700	2.9548320
H	-7.1103170	1.0984880	2.9449200
C	-3.7418830	-0.9182880	-2.6512190
H	-4.0560140	-1.9419700	-2.4104020
H	-2.9354240	-0.9791060	-3.3825930
H	-4.5881410	-0.4200000	-3.1394670
C	3.5902240	-2.5132190	-0.4946890
H	4.3893800	-1.9640030	-0.9955110
H	3.9940480	-3.5074210	-0.2569270
C	4.4070740	-1.0554170	1.2784060
H	5.3389400	-1.5687510	1.0042280
H	4.3845060	-1.0750690	2.3741970
C	0.4324590	-1.6464300	-2.4456170
H	0.1614530	-0.7451120	-2.9986520
H	0.5340060	-2.4527020	-3.1848130
C	5.7741040	1.1256060	1.1132400
H	5.5867810	2.0397270	1.6843170
H	6.2368080	1.4312190	0.1680090
H	6.4807010	0.4968670	1.6604520
C	-0.6819650	-2.0051670	-1.4606460
H	-1.5526450	-2.3466140	-2.0355010
H	-0.3823740	-2.8633070	-0.8559990
C	-1.5522640	-1.3180550	0.7451860
H	-2.0382490	-0.4550620	1.2036950

H	-2.3357300	-2.0832300	0.6342880
C	2.5694660	-2.6015570	1.7611800
H	1.9515780	-3.3457700	1.2554960
H	3.2977800	-3.1607020	2.3652390
C	0.2179530	0.1741290	2.7988370
H	-0.5391560	0.7564920	2.2746080
H	1.0708660	0.8278010	2.9911950
H	-0.1932020	-0.1718710	3.7567220
C	-0.4961910	-1.8841900	1.6936940
H	-0.1015500	-2.8204380	1.2954820
H	-0.9910960	-2.1333400	2.6432820
C	2.4070290	-2.6805870	-1.4471200
H	1.6562390	-3.3301390	-0.9944070
H	2.7566680	-3.1871680	-2.3573550
C	1.7161130	-1.7336050	2.6883450
H	2.3517520	-1.0039510	3.1951890
H	1.2678840	-2.3613310	3.4696820
C	2.6039510	-0.6158090	-2.7436030
H	2.1276180	0.3439800	-2.9505950
H	2.7425140	-1.1637860	-3.6852440
H	3.5812810	-0.4191330	-2.3025860
C	3.6618590	4.9343540	-0.7615750
H	4.4776990	4.5081600	-1.3531680
H	4.0072830	5.0382900	0.2736990
H	3.4192710	5.9225910	-1.1564960

Table S31. Cartesian coordinates for the Zn^{2+} Δ(λ,λ,λ,λ) isomer.

E(RB3LYP) = -3909.803106 Hartree.

Atom	X	Y	Z
Zn	-1.6044620	-0.2335760	-0.0901700
S	7.9904230	0.0636100	0.2113720
S	-0.8901060	1.7246600	-1.3170210
N	-3.3101750	-1.5993820	0.9275360
N	-3.3942730	1.0464900	0.1330360
N	5.4509330	-0.1886870	-0.5309360
H	5.8230980	-0.9597520	-1.0788960
N	5.8350900	1.4903430	0.9960240
H	4.8315870	1.6316900	0.9207570
N	-0.8731710	-0.2847970	2.0255360
N	-3.4020410	2.3529960	-0.3138890
N	-1.5958760	-2.0337000	-1.4574300
N	0.9940500	-0.9548650	-0.2418200
N	4.1150630	0.1128930	-0.4926250
C	6.3472410	0.5008910	0.2512940
N	-2.2532430	4.0035210	-1.4028810

H	-1.4656130	4.2494480	-1.9860830
C	3.3089860	-0.5351320	-1.2663820
C	-4.4850070	0.6141080	0.6679290
C	1.8388960	-0.1425050	-1.1539180
H	1.3916310	-0.1632150	-2.1507850
H	1.7899930	0.8890120	-0.8025740
C	-2.3098390	2.7160210	-0.9608970
C	6.6074970	2.3454810	1.8898080
H	7.3763420	2.8947680	1.3390770
H	5.9198960	3.0567900	2.3490020
H	7.0927110	1.7578540	2.6744780
C	3.7575780	-1.5837410	-2.2576130
H	4.1417480	-2.4807550	-1.7555860
H	2.9401940	-1.8898580	-2.9110870
H	4.5600050	-1.1961640	-2.8977460
C	-3.5442330	-2.7198080	-0.0114860
H	-4.2883310	-2.3940830	-0.7397660
H	-3.9657290	-3.5952910	0.5031670
C	-4.5362030	-0.8099520	1.1696310
H	-5.4035920	-1.3007140	0.7109230
H	-4.7684360	-0.7838850	2.2422640
C	-0.2108060	-2.4424650	-1.8357600
H	0.1022880	-1.8009180	-2.6615090
H	-0.2210940	-3.4703440	-2.2229300
C	-5.7277730	1.4434230	0.8321890
H	-5.5149310	2.3389650	1.4246480
H	-6.0895600	1.7867200	-0.1426720
H	-6.5198460	0.8725220	1.3229870
C	0.7978250	-2.3459150	-0.6903400
H	1.7339410	-2.8225520	-1.0106820
H	0.4511180	-2.9366170	0.1596300
C	1.4507620	-0.8474690	1.1560480
H	1.8441310	0.1605600	1.2994620
H	2.2827760	-1.5332850	1.3764820
C	-2.6776640	-2.0239350	2.1951460
H	-1.9701900	-2.8263630	1.9799370
H	-3.4183650	-2.4414870	2.8918000
C	-0.5961850	1.1121540	2.4499740
H	0.1945220	1.5422120	1.8354700
H	-1.4947200	1.7170330	2.3143350
H	-0.2933240	1.1501130	3.5051830
C	0.3487990	-1.1254160	2.1794320
H	0.0476490	-2.1727730	2.1276630
H	0.7726420	-0.9744610	3.1826050
C	-2.2707480	-3.1506230	-0.7377950
H	-1.5621970	-3.5749870	-0.0250290
H	-2.5251220	-3.9532270	-1.4439010

C	-1.9674480	-0.8495290	2.8676700
H	-2.6859090	-0.0492810	3.0598390
H	-1.5747760	-1.1649580	3.8433200
C	-2.3452680	-1.6972520	-2.6990250
H	-1.8681360	-0.8456350	-3.1870180
H	-2.3635550	-2.5507070	-3.3898310
H	-3.3709540	-1.4152710	-2.4579750
C	-3.3603600	4.9469440	-1.2950430
H	-4.2504880	4.5893830	-1.8258150
H	-3.6308340	5.1139370	-0.2480480
H	-3.0425920	5.8951510	-1.7326140

Table S32. Cartesian coordinates for the Zn^{2+} Δ(δ,δ,δ,δ) isomer.

E(RB3LYP) = -3909.804712 Hartree.

Atom	X	Y	Z
Zn	-1.5991130	-0.2485660	-0.0834790
S	-1.0700020	1.7736560	-1.3026010
S	7.9616980	0.0862250	0.1812530
N	-0.5588830	-0.1375320	1.8834070
N	0.9841260	-0.9107350	-0.6519780
N	4.0934350	0.2814570	-0.5356640
N	-1.6972240	-2.1001220	-1.3401190
N	-3.4864020	0.8764200	0.1650330
N	5.4337620	0.0138140	-0.6349960
H	5.8188670	-0.5979510	-1.3495420
N	-3.0566260	-1.6036180	1.2404810
N	5.7845410	1.2570170	1.2705360
H	4.7807070	1.4068810	1.2196300
N	-3.6082480	2.1844610	-0.2563120
N	-2.6258100	3.9276940	-1.3645860
H	-1.7852910	4.3086020	-1.7763180
C	1.8241440	0.1601400	-1.2480110
H	1.3776780	0.4302270	-2.2089910
H	1.7606470	1.0393010	-0.6062720
C	1.3968000	-1.2603870	0.7204580
H	0.9951970	-2.2457380	0.9637290
H	2.4880820	-1.3499590	0.8099380
C	3.2986740	-0.1650240	-1.4519150
C	6.3142820	0.4898490	0.3078560
C	-2.5587370	2.6439630	-0.9135510
C	-2.5738500	-1.4499990	2.6334220
H	-3.0760710	-0.5822940	3.0650170
H	-2.8576250	-2.3151500	3.2489390
C	-4.5402650	0.3254330	0.6658060

C	-1.0596150	-1.2633430	2.7198200
H	-0.5561440	-2.1765020	2.3990280
H	-0.7848910	-1.1037870	3.7718610
C	0.9249780	-0.2287040	1.7465040
H	1.3719250	-0.4621700	2.7226620
H	1.2879490	0.7611110	1.4642290
C	-0.4320210	-2.8862630	-1.2684830
H	-0.3751650	-3.3484650	-0.2819790
H	-0.4722840	-3.7086630	-1.9969580
C	-4.4433570	-1.1163550	1.1110180
H	-4.9863190	-1.7244510	0.3783660
H	-4.9931820	-1.2454130	2.0536750
C	0.8371160	-2.0782650	-1.5380150
H	0.8236720	-1.7166250	-2.5680560
H	1.6858410	-2.7754640	-1.4630080
C	-2.8927410	-2.9692550	0.7006650
H	-1.9822380	-3.4017980	1.1196410
H	-3.7181750	-3.6273230	1.0063950
C	3.7595580	-0.9441210	-2.6608610
H	4.1193850	-1.9436150	-2.3851830
H	2.9539000	-1.0683860	-3.3845950
H	4.5811930	-0.4257750	-3.1700470
C	-0.9021480	1.1677690	2.5072720
H	-1.9812110	1.2590920	2.6317110
H	-0.4207360	1.2717310	3.4889360
H	-0.5684710	1.9753730	1.8535540
C	-2.8214420	-2.9383130	-0.8272800
H	-3.7484220	-2.5224830	-1.2283730
H	-2.7398570	-3.9629330	-1.2129360
C	-1.9840580	-1.7079740	-2.7455860
H	-1.1900130	-1.0681410	-3.1294170
H	-2.0772650	-2.5926870	-3.3897310
H	-2.9164740	-1.1415160	-2.7798750
C	6.5382470	1.8638660	2.3618570
H	7.3038580	2.5444710	1.9786290
H	5.8372570	2.4260940	2.9802280
H	7.0251600	1.1004490	2.9754760
C	-5.8721990	1.0090200	0.7901670
H	-6.1620400	1.4651970	-0.1606600
H	-6.6465740	0.3054410	1.1056350
H	-5.8161860	1.8181440	1.5275450
C	-3.7247020	4.8371570	-1.0598130
H	-4.6729440	4.4416350	-1.4359850
H	-3.8266680	5.0035060	0.0190290
H	-3.5215060	5.7917450	-1.5485850

Table S33. Cartesian coordinates for the Zn^{2+} $\Lambda(\lambda,\lambda,\lambda,\lambda)$ isomer.

E(RB3LYP) = -3909.804654 Hartree.

Atom	X	Y	Z
Zn	1.5751400	-0.2277190	-0.0445140
S	0.9678790	1.7784400	-1.2428200
S	-7.9243330	0.1315950	0.2602890
N	0.4708060	-0.2665040	1.8769830
N	-0.9438090	-1.0410940	-0.7269090
N	-4.0733990	0.2106950	-0.5613540
N	1.8390540	-2.0198370	-1.3582050
N	3.4191810	0.9646770	0.2195360
N	-5.4173180	-0.0523790	-0.6029080
H	-5.8250400	-0.7273660	-1.2443080
N	3.0824880	-1.5366710	1.2924690
N	-5.7101040	1.3776710	1.1775340
H	-4.7060970	1.5083300	1.0896700
N	3.5107080	2.2623280	-0.2423480
N	2.4590010	3.9753060	-1.3370000
H	1.7007330	4.2501800	-1.9459710
C	-1.8231100	0.0091310	-1.3002410
H	-1.4179210	0.2759640	-2.2804990
H	-1.7520690	0.8977530	-0.6719940
C	-1.3706170	-1.4702130	0.6178140
H	-0.9093650	-2.4346390	0.8378540
H	-2.4552990	-1.6374820	0.6720530
C	-3.2998230	-0.3350890	-1.4409130
C	-6.2704140	0.5255030	0.3079970
C	2.4413800	2.6878820	-0.8891230
C	2.5428180	-1.4614100	2.6693300
H	2.9678750	-0.5754130	3.1443970
H	2.8623190	-2.3242120	3.2708290
C	4.4864530	0.4621830	0.7428880
C	1.0175270	-1.3805280	2.7025560
H	0.5861460	-2.3154700	2.3414510
H	0.6951030	-1.2670570	3.7468390
C	-0.9998330	-0.4484120	1.6930700
H	-1.4563150	-0.7462760	2.6467360
H	-1.4207780	0.5244680	1.4339900
C	0.6259630	-2.8902100	-1.3705260
H	0.5664100	-3.4008640	-0.4083560
H	0.7480570	-3.6734240	-2.1321100
C	4.4425560	-0.9701950	1.2225450
H	5.0614610	-1.5640410	0.5397380
H	4.9467770	-1.0421170	2.1959640
C	-0.6882320	-2.1588010	-1.6518440
H	-0.6692570	-1.7555490	-2.6664860

H	-1.4862990	-2.9168790	-1.6314240
C	3.0098500	-2.8871920	0.6985180
H	2.1091240	-3.3813590	1.0665460
H	3.8567300	-3.5134570	1.0128670
C	-3.7829200	-1.2428730	-2.5461430
H	-4.1225050	-2.2093850	-2.1521420
H	-2.9945330	-1.4378060	-3.2730630
H	-4.6236660	-0.7918050	-3.0868520
C	0.7098440	1.0401940	2.5479190
H	1.7768710	1.1977560	2.7065800
H	0.1962990	1.0827160	3.5176790
H	0.3416590	1.8437350	1.9078850
C	2.9947600	-2.8038370	-0.8288900
H	3.9078910	-2.3156250	-1.1772680
H	2.9946370	-3.8164530	-1.2529840
C	2.1523730	-1.5654960	-2.7405050
H	1.3314820	-0.9676580	-3.1355110
H	2.3272640	-2.4224270	-3.4046200
H	3.0456370	-0.9384370	-2.7225150
C	-6.4311260	2.0977660	2.2211110
H	-7.1978800	2.7482860	1.7908180
H	-5.7096330	2.7079650	2.7659820
H	-6.9118890	1.4041950	2.9169490
C	5.7928480	1.1948440	0.8597900
H	6.0985880	1.5921730	-0.1127170
H	6.5779520	0.5390060	1.2439690
H	5.6903180	2.0518150	1.5347770
C	3.6297660	4.8425230	-1.2601560
H	4.4832780	4.4225670	-1.8057050
H	3.9311920	4.9982730	-0.2202810
H	3.3671560	5.8070660	-1.6986210
